# Tourist perceptions and living heritage experiences in Nanjing Tulou traditional villages, Fujian, China: A social media data analysis

**DOI:** 10.1371/journal.pone.0354702

**Published:** 2026-08-06

**Authors:** Yudong Zhang, Xiang Li, Rong Zhang, Qi Liao, Jinsong Wang

**Affiliations:** 1 College of Art and Design, Sanming University, Sanming, China; 2 College of Architecture and Engineering, Sanming University, Sanming, China; 3 School of Design, Jiangnan University, Wuxi, China; Macau University of Science and Technology, MACAO

## Abstract

This study examines how tourists perceive living heritage experiences in the Nanjing Tulou Scenic Area, a representative component of the wider Fujian Tulou World Heritage property. Drawing on Service-Dominant Logic and the distinction among place identity, place image, and place reputation, we analyzed nearly fifteen years of visitor reviews from Ctrip and Dianping using high-frequency word analysis, semantic network analysis, sentiment analysis, and stage-based topic-evolution analysis. The results show that tourists’ place image of Nanjing Tulou is shaped by three interrelated dimensions: landscape features, village impression, and service experience, with service-related issues forming the most salient dimension. While overall sentiment is predominantly positive, negative evaluations mainly concern over-commercialization, transport accessibility, ticketing, interpretation, and service management. The findings suggest that Tulou tourism is perceived not only through architectural symbols and cinematic imagery, but also through everyday encounters with resident life, commercial spaces, and tourism services. Based on these results, the study proposes a Culture-Service Symbiosis framework, arguing that service functions as both a cultural decoder and an authenticity buffer in living heritage destinations.

## 1. Introduction

Traditional villages are regarded as living cultural heritage that embodies collective memory, community identity, and the long-standing interaction between humans and nature [1–3]. Their core value is embodied not only in the physical structure of traditional architecture, but also in the continuity of daily activities and the authenticity of culture [4]. However, under the dual pressures of rural revitalization and large-scale tourism, many traditional villages have found themselves caught in the dilemma of “preservation versus development,” facing issues such as spatial homogenization, the ritualization of cultural performances, and the disappearance of local lifestyles [5].

Research on traditional rural tourism has provided valuable insights for the preservation of architectural heritage and cultural landscapes, yet three significant shortcomings remain. First, the service dimension has been overlooked. Previous studies have often focused on the intrinsic value of architectural heritage [6], while paying insufficient attention to the mediating role that service facilities and tourism infrastructure play in shaping cultural experiences and overall perceptions [7]. Second, there are methodological limitations. Most empirical studies rely on static approaches such as field surveys, questionnaires, and interviews [8–9], which struggle to capture the dynamic, multidimensional perceptual characteristics of tourists that evolve over time. Third, insufficient understanding of commercialization mechanisms. Although discussions about the negative impacts of commercialization have been extensive, empirical evidence remains scarce on how commercial development and management practices specifically undermine the perceived authenticity of living heritage sites.

To enhance the depth of the research, this study adopts a multi-method text-mining approach based on large-scale social media review data, using the Nanjing Tulou Scenic Area, a representative component of the Fujian Tulou World Heritage property in Fujian Province, China, as a case study [10–12]. Based on nearly fifteen years of online reviews from major travel platforms, this study aims to: (1) identify core spatial nodes and symbolic landscapes that capture tourists’ attention; (2) analyze key perceptual elements that shape the tourist-generated place image of Tulou villages as both architectural heritage and living communities; (3) reveal primary factors triggering negative evaluations and their impact on cultural authenticity; and (4) clarify how service encounters mediate the transformation from place identity to tourist-perceived place image. The contributions of this study are as follows: Theoretically, by introducing the distinction among place identity, place image, and place reputation and by extending Service-Dominant Logic (SDL), the study develops a Culture-Service Symbiosis framework to better understand the role of service in living heritage experience. Methodologically, it employs stage-based topic modeling and Sankey-based topic-evolution analysis to overcome the limitations of static research, providing a new analytical paradigm for monitoring heritage perceptions over extended time sequences.

## 2. Literature review

### 2.1. Living heritage and research trends in traditional villages

Traditional villages are increasingly recognized as “living heritage,” a paradigm that transcends the static preservation of physical monuments to prioritize the continuity of community life and social identity [13]. For living heritage sites such as Fujian Tulou, the core value lies not in freezing the past at a particular moment, but in sustaining everyday life, customary practices, and community activities so that heritage can be perceived and experienced in a living state. However, under the dual pressures of rural revitalization and tourism development, such sites commonly face a profound tension between conservation and development. On the one hand, tourism can promote local economic recovery and increase the social visibility of heritage; on the other hand, inappropriate development may lead to spatial homogenization, the performative commodification of culture, and the alienation of community life [14]. For example, officially imposed standardized management measures, such as prohibiting traditional incense-burning practices, may contribute to physical conservation while weakening the authenticity and integrity of community life [15]. As MacCannell’s theory of staged authenticity suggests, tourists often seek access to the “backstage” of destination life in pursuit of genuine experience, whereas tourism development frequently constructs a highly scripted “frontstage” that only appears authentic [5]. Therefore, the sustainable development of living heritage sites is not only a matter of material conservation, but also of how to sustain the authenticity of community life and balance conservation with development [14].

In recent years, research on traditional villages and living heritage has developed into a relatively rich and multidimensional framework. At the physical and spatial level, studies have moved from descriptive analysis toward the quantitative assessment of building performance, spatial order, and environmental adaptability, including measurements of the thermal comfort and climate adaptability of vernacular architecture [16–17], as well as the identification and application of traditional village spatial structure, environmental wisdom, and their digital expression through cultural landscape gene theory [18–20]. At the perceptual and experiential level, scholars have paid increasing attention to more detailed forms of interaction between visitors and place, such as visual attention revealed through eye-tracking [21], the spatio-temporal vitality of “production–living–ecological” spaces captured through Wi-Fi sensing [22–23], and the effects of natural soundscapes on landscape perception and restorative experience [24]. Building on these on-site and perceptual studies, research has further expanded beyond immediate environmental experience to broader issues of heritage communication, environmental memory, and value cognition, such as the emphasis on the authenticity of environmental memory in the concept of “weather heritage” [25], the limitations of virtual influencers in heritage communication [26], and the role of film media in shaping heritage image and enhancing conservation intentions [27]. Overall, existing studies have deepened the understanding of traditional villages and living heritage across the dimensions of architecture, space, perception, and communication, but their primary focus still remains on heritage objects themselves and the ways in which they are represented.

Despite these advances in methods and perspectives, existing studies still share a common theoretical limitation: most tend to treat traditional villages as static spatial objects, visual objects, or technical objects of analysis, rather than as dynamic and co-produced service ecosystems. In other words, current research pays more attention to architectural form, environmental performance, landscape perception, or communication media themselves, while paying much less attention to the critical interface among tourists, residents, and service processes in living heritage settings. For destinations in which everyday life itself constitutes a major attraction, cultural authenticity is not simply something to be visually observed; rather, it is continuously experienced and interpreted through daily encounters, spatial use, and service interaction [5,13]. However, existing studies remain insufficient for explaining how modern service systems intervene in living heritage experiences, and they do not fully reveal the complex relationships among commercialization, service processes, and authenticity [14]. Therefore, it is necessary to re-examine tourist perception and living heritage experience in traditional villages from the perspective of the interaction between culture and service, so as to explain how service processes intervene in cultural understanding, authenticity judgment, and destination image formation, and to provide theoretical clues for the subsequent analysis.

### 2.2. Multidimensional analysis of tourist perception and experience

Tourists’ perceptual experiences in traditional villages are multidimensional constructs that serve as important indicators of a destination’s appeal and experiential quality. Recent systematic reviews have conceptualized this experience through four interconnected dimensions: cultural authenticity, environmental adaptability, emotional experience, and service facility effectiveness, noting that significant perceptual tension often exists among these elements [28–29].

At the core of this multidimensional experience is cultural authenticity, which has been shown to be a key factor in enhancing visitors’ willingness to protect heritage [27]. This perception of authenticity extends beyond architectural form to encompass psychological compatibility, natural ecology, and narrative continuity [30]. Specifically, “authenticity seekers” exhibit heightened sensitivity toward traditional construction techniques and the authentic unfolding of local lifestyles [31].

Recent empirical studies have identified a recurring “cognitive tension” between cultural appreciation and service delivery in heritage sites. While visitors often report highly positive emotional responses toward static architectural heritage (e.g., yielding up to 57.44% positive sentiment), their satisfaction with dynamic service facilities frequently remains disproportionately low, creating a mismatch that may constrain overall experience quality [32]. Although service effectiveness significantly and positively influences overall satisfaction, practical service supply often falls short of tourist expectations [33]. Understanding this tension is important, as effective service delivery is closely associated with emotional experiences, particularly place attachment. Studies show that memorable tourism experiences and strong place attachment serve as important bridges linking perceptions of authenticity with long-term revisit intentions [34]. Moreover, fostering strong place identification significantly stimulates tourists’ behavioral intentions to participate in ecological and cultural conservation [35].

Therefore, the relationship between service facility effectiveness and cultural authenticity deserves closer theoretical attention, as it helps shape how traditional village environments are experienced as living heritage.

### 2.3. Evolution and applicability of social media data methods

Social media data has increasingly been used as a dynamic spatio-temporal source for examining landscape perception and heritage value. Research paradigms have gradually evolved from simple textual frequency analysis toward approaches such as multimodal learning, spatio-temporal analysis, and physiological measurement, thereby expanding the ability to interpret the complex characteristics of cultural heritage sites such as traditional villages and vernacular architecture. In the early stages of research, image deconstruction of cultural heritage sites was a mainstream application. To address management challenges such as overtourism and commercialization in heritage sites, scholars used geotagged photographs to identify visitor preference hotspots for specific landscape combinations [10]. High-frequency word analysis was also employed to quantify visitors’ perceived imagery. For instance, an analysis of Macao’s World Cultural Heritage sites identified crowding and commercialization as important sources of negative evaluation [36], illustrating the usefulness of social media data in examining heritage perception. This phase also suggested that, particularly in data-scarce regions, social media data can serve as a useful complement to traditional questionnaires [37].

With the integration of artificial intelligence techniques, research has increasingly shifted toward the quantitative examination of micro-level characteristics of traditional village environments and vernacular architecture. In text mining, natural language processing (NLP) has been widely applied to capture implicit spatial issues that are difficult to identify through conventional questionnaires. Shen et al. (2024) used Word2Vec to reveal the discrepancy between landscape attention and visitor satisfaction in traditional villages [38], while Liu et al. (2025) combined NLP with GIS to identify underutilized spaces and priority renewal zones through sentiment scoring [39], In image-based studies, computer vision methods have been used to quantify the complex textures of vernacular architecture. Tu et al. (2025) and Meng et al. (2024) applied semantic segmentation models to panoramic images to extract indicators such as green visibility and sky openness in traditional alleyways, enabling the development of models predicting visitors’ willingness to linger [40–41]. Such approaches improve the measurement of micro-spatial quality in vernacular environments compared with manual surveys.

Current research is increasingly shifting toward multi-source data integration combining digital humanities, spatial analysis, and physiological indicators in order to better understand human–village interaction. By integrating social media data with Geographic Information Systems (GIS), drone-based reality modeling, and related technologies, recent studies have attempted to assess overall spatial vitality and landscape genetic characteristics in a more integrated manner [42]. Scholars have also explored the fidelity of heritage perception in emerging media contexts. For instance, Fei et al. (2025) examined the role of 720-degree panoramic 3D technology in reconstructing perceptions of authenticity in traditional villages [43], while Ren et al. (2025) combined electroencephalogram (EEG) data with street-scene features to investigate the nonlinear influence of rural environments on neurophysiological activity [44].

However, despite these methodological advances, an important limitation remains. Most of these approaches are still cross-sectional and spatially static, making it difficult to trace how visitor perceptions evolve over longer periods. In addition, they often pay insufficient attention to the mediating role of dynamic service interactions in shaping living heritage experiences. As a result, the specific relationship among commercialization, service processes, and authenticity remains insufficiently examined. Consequently, the combination of large-scale social media text and stage-based topic modeling and Sankey-based topic-evolution analysis is well suited to the present study. The large sample size and 15-year time span of the UGC dataset make it possible to trace changes in visitor perception that are difficult to capture through static surveys or cross-sectional spatial analysis. By identifying recurring themes related to service friction and commercialization in large-scale unstructured reviews, this longitudinal approach provides a useful basis for examining the tension between preservation and development in living heritage sites.

### 2.4. Place Identity, Place Image, and Place Reputation

To avoid conceptual overlap among tourist perception, destination image, cultural authenticity, and heritage attractiveness, this study further distinguishes place identity, place image, and place reputation. Place identity refers to the relatively enduring material, cultural, social, and symbolic characteristics through which a place is recognized and differentiated. Place image refers to the selective and subjective representation of a place formed by individuals or groups through perception, experience, media exposure, and communication. Place reputation is a more generalized and accumulated public evaluation that emerges over time through repeated images, institutional recognition, word of mouth, and wider destination branding processes [45–46].

This distinction is particularly important for the Nanjing Tulou case. The place identity of the wider Fujian Tulou heritage landscape includes rammed-earth architecture, settlement morphology, clan organization, resident everyday life, ritual and customary practices, mountain landscape settings, and World Heritage status. The object measured in this study is not this full identity itself, but the place image reconstructed from tourist review texts on Ctrip and Dianping. This image is selective and mediated by visitors’ expectations, film and animation narratives, service encounters, transportation experiences, and perceptions of commercialization. Place reputation, in turn, refers to the broader and more generalized public evaluation of Fujian Tulou and Nanjing Tulou as a World Heritage attraction, a cinematic tourism destination, and, in some reviews, an over-commercialized tourist site.

Accordingly, the present study examines tourist-generated place image rather than claiming to measure the complete identity or reputation of Nanjing Tulou. The analytical focus is on how selected aspects of identity are perceived, filtered, and evaluated in online reviews, and how service encounters influence this transformation. This clarification also provides the conceptual basis for the Culture-Service Symbiosis framework proposed below: service is treated as a mediating interface through which latent cultural resources are decoded into visitor-understandable experiences, and through which negative frictions may accumulate into reputational risks.

### 2.5. Theoretical Framework

Building on the above conceptual distinction, this study is informed by four complementary theoretical perspectives in tourism research: the experience economy, the tourist gaze, authenticity, and service-dominant logic. Together, these perspectives provide a framework for interpreting how visitors reconstruct the place image of living heritage sites such as Nanjing Tulou, and how service experiences shape the translation of heritage identity into visitor experience.

First, Pine and Gilmore’s experience economy theory suggests that contemporary tourists seek memorable and carefully staged experiences rather than merely consuming material goods. From this perspective, services, interpretive facilities, and on-site interactions form part of the experiential setting through which cultural meaning is displayed and co-created. In the case of Nanjing Tulou, transportation, tour guide services, pricing, and interpretive arrangements are not simply logistical supports, but important elements influencing how visitors engage with Tulou architecture, resident life, and local cultural practices.

Second, Urry’s concept of the tourist gaze emphasizes that travel experiences are shaped by socially constructed modes of seeing. In heritage tourism, visitors’ attention is often directed toward visually iconic scenes, symbolic landscapes, and culturally mediated images. This perspective helps explain how cinematic symbols, such as those associated with Big Fish & Begonia and Yunshuiyao, may shape tourists’ selective visual attention to Tulou landscapes and contribute to specific patterns of visual consumption.

Third, authenticity theory, particularly MacCannell’s distinction between the “front stage” and “back stage” [5], highlights tourists’ desire to engage with what they perceive as genuine local life, as well as the tensions that emerge when tourism development produces highly scripted environments. This perspective is especially relevant for interpreting how visitors respond to commercialization, standardized services, and other arrangements that may be perceived as intrusive or inconsistent with the living character of heritage sites.

Finally, service-dominant logic (SDL) shifts attention from a product-centered model to a service-centered one in which value is co-created through interactions among providers, residents, and visitors, rather than residing solely in physical resources [47]. SDL concepts such as value co-creation, resource integration, service ecosystems, actors, institutional arrangements, and value-in-use are especially relevant for living heritage destinations, where the meaning of architecture and local practices is activated through service encounters. Tulou buildings, resident knowledge, guided interpretation, ticketing systems, transport arrangements, and digital information together form a service ecosystem. Within this ecosystem, visitors integrate physical heritage resources with service information and personal expectations to produce value-in-use.

On this basis, this study defines the Culture-Service Symbiosis framework as a mechanism through which cultural resources and service processes jointly shape tourist-generated place image in living heritage destinations. The framework contains two interrelated mechanisms. The first is cultural decoding: services such as interpretation, guiding, signage, and digital tools help visitors understand the meanings of Tulou architecture, settlement organization, and everyday community life. The second is authenticity buffering: transparent, efficient, and culturally sensitive management reduces the negative impact of commercialization, crowding, and service friction on perceived authenticity. Conversely, poor service and excessive commercialization may distort the transformation from place identity to place image and gradually weaken destination reputation.

Taken together, these theoretical perspectives guide the analytical design of this study. The analytical procedures used in this study – including high-frequency word analysis, semantic network analysis, sentiment analysis, and stage-based temporal topic analysis – are employed as empirical tools for examining how gaze, authenticity, and service-mediated experience are reflected in visitor narratives about living heritage [48]. The longitudinal social media design is therefore suited to tracing changes in tourist-generated place image over time, while remaining cautious about not equating online review texts with the full identity or reputation of the destination.

## 3. Materials and Methods

### 3.1. Research Location

**Fig 1:**
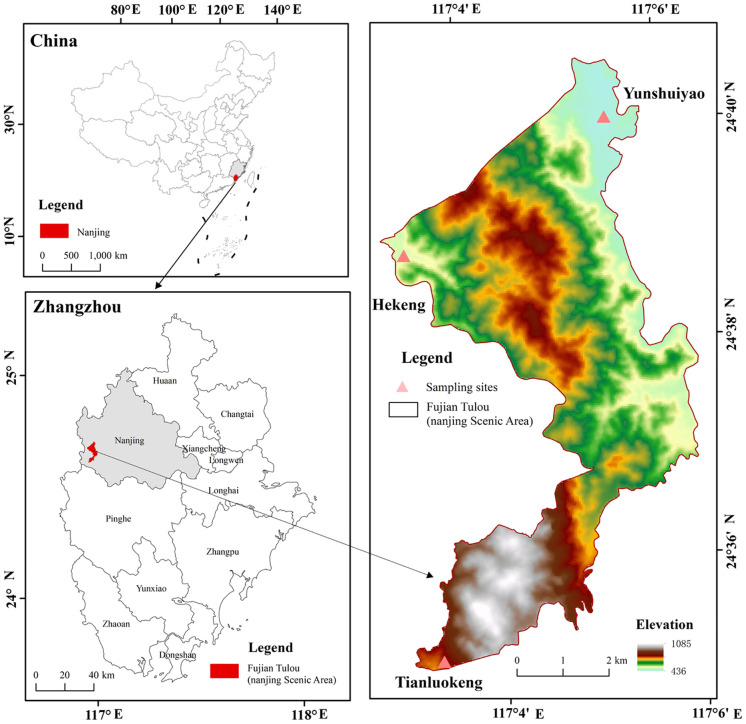
Location of the study area and sampling sites in the Nanjing component of the Fujian Tulou World Heritage property, Nanjing County, Fujian Province, China. The left panels show the location of the study area within China and Zhangzhou City, respectively, and the right panel shows the boundary of the Fujian Tulou (Nanjing Scenic Area), the sampling sites, and the elevation background. The base map was created using ArcGIS 10.8. Administrative boundary data were obtained from Natural Earth (public domain), and detailed spatial data for the Nanjing Scenic Area were derived from OpenStreetMap. Contains information from OpenStreetMap and OpenStreetMap Foundation, which is made available under the Open Database License.

This study focuses on the Nanjing Tulou Scenic Area in Nanjing County, Zhangzhou City, Fujian Province, China (Fig 1). More broadly, tulou buildings in Nanjing County are mainly concentrated in the mountainous areas of western Nanjing, especially in townships such as Shuyang and Meilin, which provide the broader geographical context of this study. Within this broader setting, the present research focuses specifically on the Nanjing Tulou Scenic Area, which is organized into three core tourist zones: the Tianluokeng Tulou Cluster, Yunshuiyao, and the Hekeng Tulou Cluster [49]. The study area is part of the wider Fujian Tulou World Heritage property rather than the entirety of that property, and forms a living cultural landscape that integrates vernacular architecture, settlement life, resident practices, and tourism services.

The selection of this study area is based on three considerations. First, the Nanjing Tulou Scenic Area has strong heritage representativeness. As part of the Fujian Tulou World Heritage property, the Nanjing component includes the Tianluokeng Tulou Cluster, the Hekeng Tulou Cluster, Huaiyuan Lou, and Hegui Lou, with a total of 20 tulou structures [50]. These buildings reflect the long-term development of tulou vernacular residential architecture and provide an appropriate setting for examining the livingness of heritage in relation to architecture, settlement form, resident everyday life, clan organization, and ritual or customary practices [6].

Second, the study area is suitable for examining the tension between heritage conservation and tourism development. The three core tourist zones—Tianluokeng, Yunshuiyao, and Hekeng—present different combinations of architectural heritage, village life, natural landscape, and tourism services. This makes the area appropriate for analyzing how visitors perceive authenticity, commercialization, and service quality in a living heritage destination.

Third, the Nanjing Tulou Scenic Area provides a relatively rich source of long-term user-generated content. As a well-known heritage tourism destination, it has accumulated a substantial number of visitor reviews on major online travel platforms over an extended period. This makes it possible to trace changes in tourist perception over time and supports the use of large-scale text analysis in this study.

### 3.2. Data collection and filtering

This study focuses on the Fujian Tulou (Nanjing) scenic area, targeting core keywords such as “Nanjing Tulou,” “Tianluokeng Tulou Cluster,” and “Yunshuiyao.” To capture the longitudinal evolution of tourist perceptions across temporal and generational variations, a custom Python crawler (v3.12.3) was used to collect online review text data from two major Chinese travel platforms: Ctrip (www.ctrip.com) and Dianping (www.dianping.com). The data collection spanned an exact 15-year period from January 2010 to December 2024, yielding an initial dataset of 20,143 original reviews.

During the text filtering process, only reviews containing interpretable descriptions of the tourism experience were retained, particularly those addressing dimensions such as the scenic area environment, cultural experiences, and service quality. A multi-stage data cleansing protocol was implemented to exclude non-substantive content, removing advertisements, duplicate check-ins, images, memes, and extremely short texts. Following this rigorous filtering, a final corpus of 20,102 valid reviews was established for analysis.

To improve transparency and reduce ethical risk, data collection was limited to publicly accessible review pages. Furthermore, the data collection and analysis methods strictly complied with the terms and conditions of the source platforms (Ctrip and Dianping). Low-frequency requests with randomized delays were used during collection to avoid imposing unnecessary burden on the host platforms. The analytical dataset did not retain usernames, profile images, or precise personal location information, and all results are reported only in anonymized and aggregated form. The data were used solely for non-commercial academic research. As the authors’ institution, Sanming University, does not maintain a formal ethics review committee, this study was officially referred by the College of Arts and Design, Sanming University, to the Medical Ethics Committee of the First Hospital of Sanming City for ethical review. The project was granted exemption from ethical review by the Medical Ethics Committee of the First Hospital of Sanming City (Document No.: Lunshen Mian (2026) No. 02) on March 31, 2026. Ethics filing for the project was completed, and the exemption notice serves as the official document certifying ethical compliance. Furthermore, to support reproducibility, the core Python logic and the anonymized minimal dataset have been deposited in a public repository.

### 3.3. Research methods and objectives

**Fig 2:**
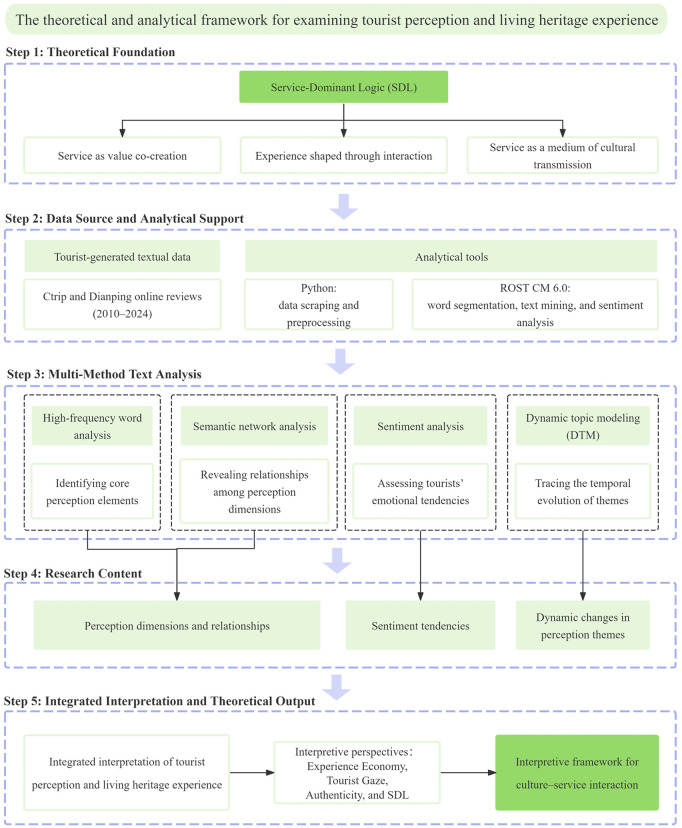
Research Framework Diagram.

This study adopts a multi-method text-mining approach to examine the core characteristics of tourist perceptions at the Nanjing Tulou and their temporal evolution. To maintain a clear analytical structure, the research design is organized into a framework linking data sources, analytical methods, research content, and interpretive perspectives (Fig 2). The research process primarily encompasses the following three dimensions.

First, in data processing and preprocessing, this study combines Python and ROST CM 6.0 to handle the large-scale review corpus collected over a fifteen-year period. Python 3.12.3 was used for data extraction, cleaning, and corpus preparation, while ROST CM 6.0, developed by Wuhan University, was used for Chinese word segmentation and high-frequency word extraction [51–53]. During the preprocessing phase, standard stop-word filtering was applied to systematically remove meaningless particles, repetitive emojis, and redundant tourism jargon (e.g., “very good,” “came here”). This step helps reduce non-substantive noise in the corpus and improves the interpretability of subsequent textual analysis.

Second, based on the cleaned corpus (N = 20,102), this study conducts a multidimensional analysis of tourist perception. It employs several complementary methods, each serving a distinct analytical purpose in examining tourist perception. High-frequency word analysis is used to identify the main objects of tourist attention and highlight key elements in visitor narratives [54–55]. Semantic network analysis is used to examine structural relationships among perception dimensions, particularly the connections between cultural elements, service-related nodes, and visitor evaluations [56–57]. Sentiment analysis is further applied to assess tourists’ emotional responses to the destination [58–59]. By examining the polarity of reviews, this method helps identify recurring sources of dissatisfaction, such as commercialization and management issues.

Third, to explore the dynamic trajectory of visitor perceptions, this study adopts a longitudinal perspective and divides the 2010–2024 period into three development phases. The Initial Development Phase (2010–2015) is characterized primarily by post-World Heritage policy guidance and infrastructure construction. The Media-Driven Growth Phase (2016–2019) corresponds to a period during which film and media exposure became more prominent in shaping destination image such as “Cloud and Water Ballad” and “Big Fish & Begonia.” Finally, the Consolidation and Pressure Phase (2020–2024) encompasses the impact of the global pandemic, the subsequent surge in regional self-driving tours, and heightened visitor sensitivity to service quality and commercial overcrowding.

To trace thematic shifts across the three developmental phases, stage-based temporal topic modeling was implemented using phase-specific topic models in the Python-based Gensim library. This procedure is not a strict continuous-time Dynamic Topic Model (DTM); rather, it estimates separate phase-specific topic models and links adjacent phases through inter-phase cosine similarity values. Topic coherence was first evaluated separately for each phase in order to determine the appropriate number of topics. The number of iterations was set to 200 for all phase-specific models. Based on the coherence evaluation, the optimal topic numbers for the three phases were identified as 10, 7, and 8, respectively. Topic modeling was then conducted separately for each phase using these settings. To examine topic evolution across time, pairwise cosine similarities were calculated between topics from two adjacent phases, and topic links were established on the basis of these inter-phase similarity values. Sankey diagrams were subsequently constructed to visualize the flow and evolution of these core themes across phases, with a similarity threshold of 0.1 applied to retain meaningful links. These procedures provide an empirical basis for examining long-term changes in tourist attention, emotional evaluation, and service-related perception in the Nanjing component of the Fujian Tulou World Heritage property.

### 3.4. Data Reliability, Representativeness, and Bias Control

As with other studies based on user-generated content, this research needs to address issues of data representativeness and methodological reliability.

First, Ctrip and Dianping were selected because they provide relatively stable and continuous review data over a long time span. As two major Chinese travel platforms with substantial textual archives, they offer better longitudinal coverage than newer short-video or lifestyle platforms such as Douyin or Xiaohongshu, which are less suitable for tracing long-term changes in visitor perception through textual records.

Second, the sample inevitably reflects the characteristics of active online reviewers who are willing to share travel experiences on digital platforms. Because explicit demographic metadata are not available for most reviews, this study uses text-derived behavioral proxies to make cautious inferences about sample tendencies. For example, frequent references to “driving” and “parking” suggest a strong presence of self-driving tourists, while expressions such as “graduation trip” or “photography” may indicate the participation of younger and more experience-oriented visitors. In addition, online review datasets often overrepresent polarized opinions, especially highly satisfied or highly dissatisfied responses. Therefore, the findings should be understood as reflecting the perceptions of active online reviewers rather than a full demographic representation of all visitors to Nanjing Tulou.

To address the representativeness risk associated with relying on domestic Chinese platforms, a supplementary international-facing platform triangulation step was conducted. Publicly visible English-language reviews on platforms such as Tripadvisor and Trip.com were read as contextual evidence rather than incorporated into the main quantitative corpus [60–61]. At the time of checking, the Tripadvisor page for Tulou de Fujian listed 155 reviews, while the Trip.com page for Tianluokeng Tulou Cluster listed more than 4,000 reviews. This lightweight comparison suggested that international-facing platform content also emphasizes architectural distinctiveness, World Heritage value, transport difficulty, guided interpretation, and commercialization. However, these pages mix international tourists, domestic users, platform-generated travel notes, and machine-translated content, and they do not provide the same comparable long-term metadata as the Ctrip-Dianping corpus. Therefore, the main statistical results remain based on Ctrip and Dianping, while the international-facing material is used only to qualify the interpretation and scope of the conclusions.

Third, to ensure the methodological reliability of the text mining outputs generated by ROST CM 6.0 and Python, strict bias control measures were implemented. During the preprocessing phase, the corpus was rigorously cleansed of advertisements, duplicate check-ins, emojis, and meaningless digital tags to optimize data quality. Furthermore, the interpretations drawn from high-frequency words, sentiment analysis, and semantic networks were continuously cross-validated against the authors’ field observations and established literature on heritage destination image [62]. Collectively, these rigorous steps minimize algorithmic artifacts and bolster confidence that the extracted themes represent genuine trajectories of visitor perception.

## 4. Results

### 4.1. High-frequency word analysis

**Fig 3:**
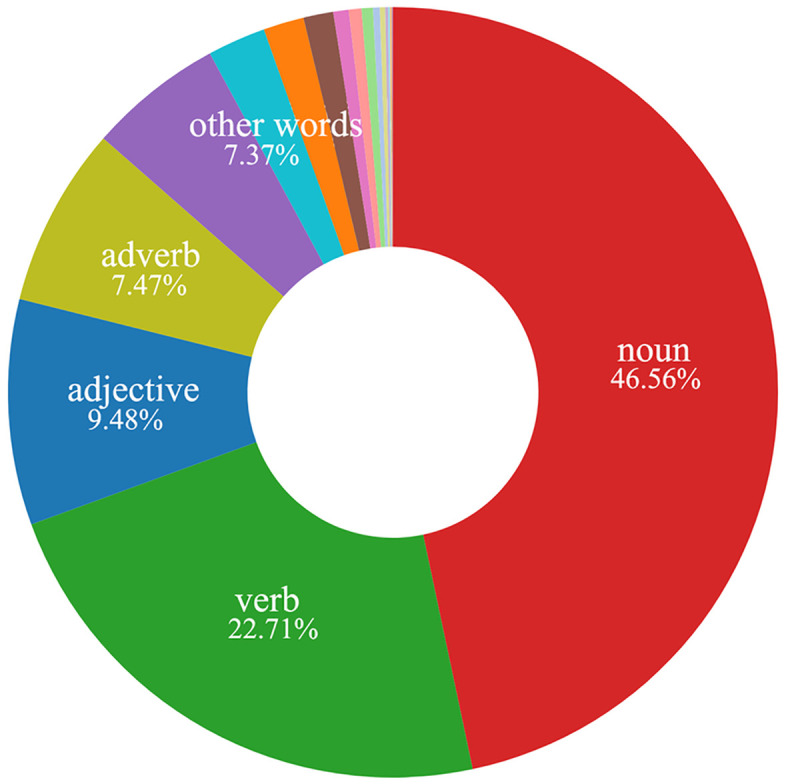
Proportional distribution of part-of-speech in high-frequency words.

**Fig 4:**
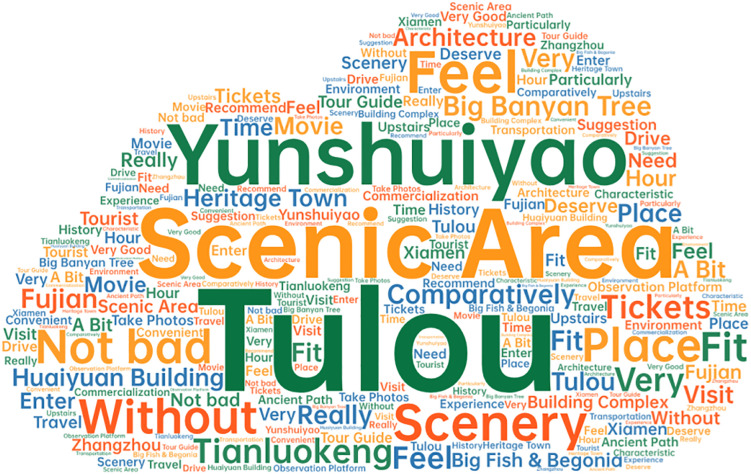
Core themes of high-frequency words.

This study utilized ROST CM 6.0 software to extract the top 50 high-frequency lexical items (Table 1, Fig 3, Fig 4), whose attributes primarily encompass four dimensions: landscape features, recreational behaviors, emotional perceptions, and environmental perceptions.

**Table 1:** High-frequency words list statistics.

Ranking Order	High-frequency words	Frequency	Part of speech	Number	Ranking Order	High-frequency words	Frequency	Part of speech	Number
1	Tulou	17445	Noun	8448	26	Zhangzhou	1812	Noun	1527
2	Scenic Area	11326	Noun	6670	27	particularly	1514	Adverb	1250
3	Yunshuiyao	10145	Noun	5376	28	hour	1416	Noun	1207
4	Feel	4832	Verb	3693	29	take photos	1345	Verb	1150
5	Scenery	3729	Noun	3376	30	tourist	1331	Noun	1110
6	Without	3239	Verb	2627	31	time	1323	Noun	1111
7	Not bad	3121	Adjective	2722	32	recommend	1244	Verb	1086
8	Place	3109	Noun	2617	33	very good	1199	Adjective	1093
9	Tianluokeng	3063	Noun	1916	34	enter	1199	Verb	973
10	Very	2879	Adverb	2184	35	Visit	1165	Noun	984
11	Tickets	2806	Noun	2315	36	observation platform	1134	Noun	796
12	Big Banyan Tree	2514	Noun	1977	37	Upstairs	1133	Verb	936
13	Comparatively	2477	Adverb	1972	38	Generally	1128	Adjective	1021
14	Fujian	2443	Noun	1844	39	commercialization	1103	Noun	1050
15	Really	2307	Adverb	1814	40	environment	1081	Noun	1005
16	Huaiyuan Lou	2130	Noun	1646	41	History	1072	Noun	932
17	Architecture	2129	Noun	1598	42	ancient trails	1052	Noun	737
18	Film	2110	Noun	1695	43	fit	1011	Verb	943
19	Heritage Town	2048	Noun	1397	44	experience	993	Noun	894
20	Big Fish & Begonia	1996	Noun	903	45	convenient	990	Adjective	939
21	Drive	1939	Verb	1621	46	Need	988	Verb	847
22	Deserve	1924	Verb	1858	47	transportation	944	Noun	859
23	Building Complex	1908	Noun	1227	48	characteristic	912	Noun	832
24	Tour Guide	1843	Noun	1402	49	travel	910	Verb	824
25	Xiamen	1824	Noun	1520	50	certainly	905	Adverb	797

Source: Author statistics.

Analysis reveals that landscape-specific keywords hold a central position in visitor perceptions. Beyond foundational geographic identifiers like ‘Tulou’ and ‘Tianluokeng’, terms closely tied to renowned film and television works, such as ‘Yunshuiyao’, ‘Big Banyan Tree’, and ‘Huaiyuan Tulou’, appeared frequently. This suggests that film- and television-related imagery plays an important role in reshaping destination image and directing visitor attention through visual symbolism.

In terms of tourist activities, “self-driving,” “photography,” and ‘visiting’ stand out most prominently. This not only reflects the current preference for independent travel among tourists but also reveals a visual consumption pattern driven by “film and television check-ins”: visitors tend to recreate movie scenes through their lenses to validate and experience cultural heritage.

Regarding emotional and environmental perceptions, evaluations generally show positive tendencies, with visitors commonly describing the unique charm of Hakka architecture and culture as “impressive” and “exceptional.” However, the frequent emergence of “commercialization” as an environmental perception term reveals underlying tensions: while enjoying tourism conveniences, visitors remain highly sensitive and vigilant toward excessive commercial development eroding the authenticity of the villages.

This study employs high-frequency word quantitative analysis to investigate tourists’ perceptions of the destination image of Nanjing Tulou. Findings reveal that visitor evaluations center on eight core keywords, which can be structurally categorized into three perceptual dimensions based on their attributes (Table 2).

**Table 2:** Coding analysis of tourist perception dimensions.

Core Features	One-Level Coding	Secondary Coding	Keyword Frequency	Percentage%	Keyword Frequency	Percentage%
Exhibition Layer of the “Static Heritage”	Landscape Features	Cinematic Cultural Landscape	1872	6.24%	6784	22.62%
		Architectural Heritage	3136	10.46%		
		Natural landscapes	1776	5.92%		
Perception Layer of the “Living Community”	Village Impressions	Rural life	4019	13.40%	9323	31.08%
		Environmental Quality	5304	17.68%		
Core Media Layer for Cultural Transmission	Service Experience	Accommodation and meals	5676	18.92%	13888	46.30%
		Traffic and Parking	3967	13.23%		
		Charges and Management	4245	14.15%		

First is the Landscape Features dimension (22.62%), comprising three subcategories: “Cultural Landscape,” “Architectural Heritage,” and “Natural Landscape.” Second is the Village Impression dimension (31.08%), consisting of “Living Atmosphere,” which reflects authentic local characteristics, and “Environmental Quality.” Crucially, the Service Experience dimension commands the absolute highest perceptual weight at 46.30%, encompassing operational elements such as “accommodation and dining experiences,” “transportation and parking,” and “scenic area fees and management.”

This structural distribution, particularly the prominence of service-related elements (46.30%), suggests that service plays an especially important role in how visitors evaluate Nanjing Tulou. The pattern is broadly consistent with the relevance of Service-Dominant Logic (SDL) in heritage tourism, in that visitor experience appears to be shaped not only by physical landscapes but also by service encounters and management processes. From a practical perspective, this result indicates that destination management should pay closer attention to service-related friction points, such as parking, transportation, and fee management, when seeking to improve the overall heritage experience.

### 4.2. Semantic network structure and perceptual logic

**Fig 5:**
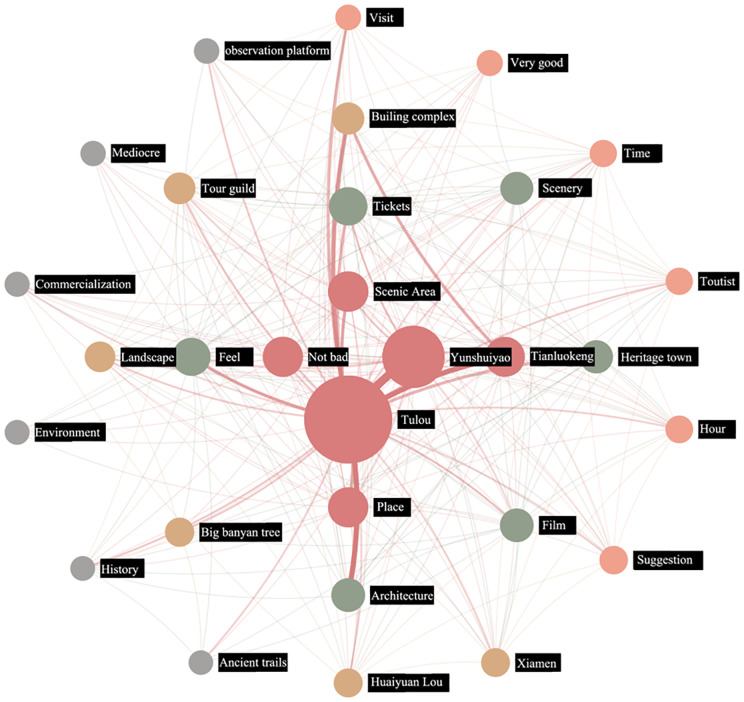
Semantic analysis of the high-frequency word network.

The semantic network constructed using ROST CM 6.0 (Table 3 and Fig 5) exhibits a distinct “core-periphery” concentric structure. This framework transcends mere lexical aggregation, instead mapping the psychological cognitive journey of visitors from superficial observation to profound experience.

**Table 3:** Structural Levels and Theoretical Connotations of the Semantic Network.

Semantic Level	Representative Keywords	Perception Logic & Theoretical Implication
Core Layer	Tulou, scenic area, scenery, Tianluokeng, place	**Visual Symbols:** The primary focal points capturing visitors’ gaze, representing the most recognizable landscape entities.
Sub-core Layer	Architecture, Film, Ancient Towns, Building Complexes	**Cultural Decoding:** Visitors construct deep cultural understanding of heritage sites through architectural entities and film/TV IP symbols.
Intermediate Layer	Tour guide, Time, Recommendations, Admission tickets, Sightseeing	**Service as Medium:** Service touchpoints—such as guided tours and ticketing—may serve as an important bridge through which static heritage is translated into dynamic visitor experience, in a way that is broadly consistent with Service-Dominant Logic.
Peripheral Layer	Commercialization, Environment, General, History, Ancient Trail	**Authenticity Tension:** Reveals tourists’ critical reflection on excessive commercialization and environmental disorder in the “front stage” while seeking the “backstage” authentic life.

First, the core layer is dominated by “Visual Symbols.” High-density nodes at the network center—such as “earth buildings,” “scenic areas,” “Tianluokeng,” and ‘landscapes’—form the cornerstone of visitor perception. This pattern is consistent with Urry’s concept of the tourist gaze, which emphasizes the centrality of visually recognizable symbols in early tourist attention [63].

Secondly, the intermediate layer embodies the function of service as a cultural medium. Following the core layer are nodes such as architecture, film, tour guides, time, and advice. This co-occurrence relationship reveals the path of deepening visitor cognition: visitors attempt to decode architectural meanings or locate film-related narrative scenes through service touchpoints such as guide explanations, route planning, signage, and time management. This pattern is consistent with SDL, because service encounters integrate heritage resources, provider knowledge, resident context, and visitor expectations into value-in-use.

Finally, the peripheral layer reveals the tension in seeking “Backstage Authenticity.” Terms like “commercialization,” “tickets,” “environment,” and “general,” situated at the network’s periphery yet closely linked to core nodes, constitute the critical dimension of perception. Drawing on MacCannell’s theory of stage authenticity [5], these nodes reflect the obstacles tourists encounter when attempting to traverse the commercially constructed “frontstage” to access authentic Hakka life (the backstage). The strong semantic link between ‘commercialization’ and “scenic area” in the network visually illustrates how commercial development disrupts perceptions of cultural authenticity, explaining the primary source of negative evaluations.

### 4.3. Tourist Emotional Preferences and Experience Pain Points

**Fig 6:**
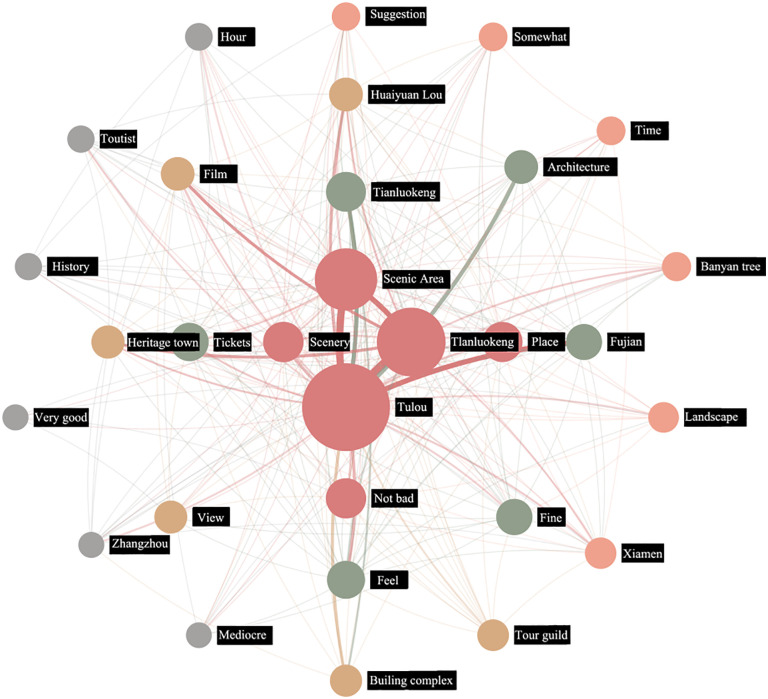
Sentiment analysis network diagram. Different colors are used solely to distinguish terms at different levels.

Sentiment analysis based on ROST CM 6.0 reveals (Table 4 and Fig 6) that visitors’ overall evaluation of Nanjing Tulou is predominantly positive (74.83%). This high satisfaction level, as reflected in semantic co-occurrence, primarily centers on two core attractions: the unique aesthetic value of the tulou architectural complex and the cultural emotional resonance evoked by film and television IPs such as “Yunshuiyao” and “Big Fish & Begonia.”

**Table 4:** Data Chart of emotion proportion.

Emotional Categories	Number of Articles	Proportion%	Emotional Segmentation Statistics	Proportion%
Positive emotions	15042	74.83	Mildly positive (0–10)	25.46
			Moderately positive (10–20)	20.23
			Strongly positive (>20)	29.14
Intermediate emotions	1671	8.31		
Negative emotions	3389	16.86	Mildly negative (−10–0)	10.81
			Moderately negative (−20 to −10)	3.66
			Strongly negative (<−20)	2.39
Total	20102	100		

Source: Author statistics.

However, the 16.86% of negative reviews, though a minority, provide crucial diagnostic insights for heritage management. Thematic analysis of the review texts reveals that visitor dissatisfaction is not randomly distributed but highly concentrated around three major structural pain points:

First, infrastructure barriers. Poor transportation accessibility in mountainous areas, scarce parking resources, and inadequate internal shuttle services may substantially increase visitors’ time costs and physical fatigue, making transportation a recurring theme in negative reviews. Second, trust crisis in management. Issues such as repeated ticket checks, unclear fees, inflated prices, and lagging environmental maintenance not only cast doubt on service professionalism but also undermine visitors’ perceptions of order, trust, and the overall site atmosphere. Third, erosion of authenticity. These critiques center on excessive commercialization and homogenized merchandise, arguing that the commercial atmosphere obscures resident life, clan-based memory, and everyday cultural practices, and may degrade the tourism experience into superficial photo-taking and check-ins.

### 4.4. Structural Dimensions and Comprehensive Interpretation of Tourism Destination Image

**Fig 7:**
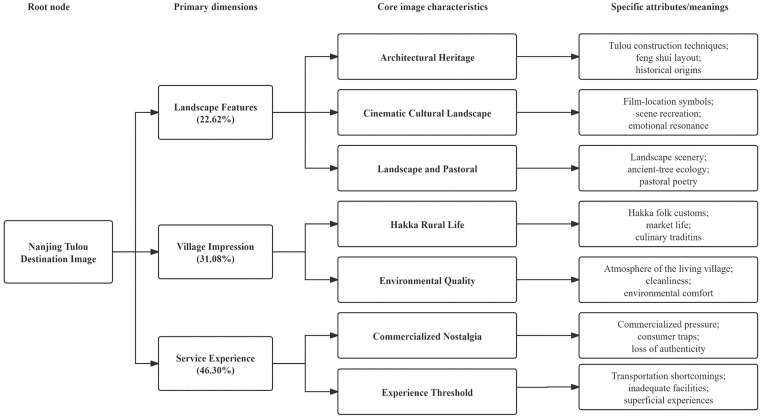
Hierarchical Coding Structure of Nanjing Tulou Destination Image. **Note:** Fig 7. Dendrogram illustrating the hierarchical relationships of destination image codings. For a detailed summary of characteristic features and representative visitor comments, please refer to Appendix A. Source: Author statistics.

Based on qualitative coding and thematic induction of the textual corpus, six key image characteristics were identified: architectural heritage value, film and television cultural influence, natural ecological ambiance, local lifestyle culture, commercialization-related experiential conflicts, and tourism service experience (see Appendix A for representative examples). To further structure these findings, a hierarchical coding process was applied. As illustrated in the resulting dendrogram (Fig 7), these characteristics do not exist independently but can be grouped into a three-dimensional perceptual structure (Tables 2 and Appendix A in S1 File).

The first dimension, Landscape Features (22.62%), includes architectural heritage, film-related scenery, and pastoral landscape elements. This dimension is associated with visitors’ visual attention to iconic scenes associated with Tulou architecture and film imagery, such as Big Fish & Begonia and Yunshuiyao, and appears to function as an important source of aesthetic appreciation and positive emotional response.

The second dimension, Village Impression (31.08%), emphasizes perceptions of local residential life, environmental atmosphere, foodways, market scenes, and community practices. This dimension may be interpreted as reflecting visitors’ interest in experiencing living settlement culture beyond curated tourism settings.

The third and most prominent dimension is Service Experience (46.30%), which includes accommodation, transportation, and management-related elements. Because this dimension accounts for the largest proportion among the three, it suggests that service-related encounters play an especially important role in shaping visitor evaluations of the destination. Rather than being viewed only as logistical support, service experience appears to be closely related to how visitors evaluate and experience the living heritage setting.

Notably, this three-dimensional structure also suggests a degree of internal tension. While the landscape dimension functions as an important pull factor and is associated with a large share of positive sentiment (74.83%), the service experience dimension concentrates a substantial proportion of negative evaluations, especially those related to commercialization and traffic congestion. This pattern suggests that the online reviews reconstruct Nanjing Tulou through a dual tourist-generated place image: on the one hand, it is associated with visually appealing and culturally symbolic heritage scenes; on the other hand, it is experienced as a living community facing practical management and service-related pressures. The findings therefore do not equate tourist image with the full identity of the place. Rather, they show how selected aspects of place identity are filtered through service encounters and may, if repeated over time, contribute to the destination’s broader reputation.

### 4.5. Topic evolution and temporal dynamics of tourist perception

**Fig 8:**
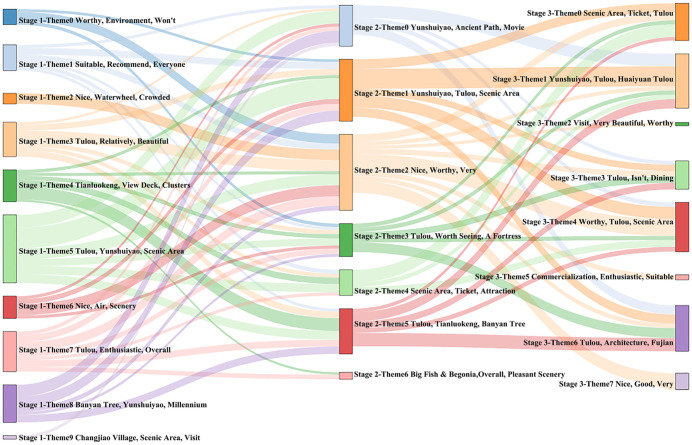
Analysis of the Spatio-Temporal Evolution of Tourists’ Perceptions.

To precisely capture the flow and shift of visitor perception focus, this study employs stage-based topic modeling and Sankey-based topic-evolution analysis to visualize thematic evolution trajectories across three developmental stages (Fig 8). The width of each node represents the theme’s weight in the current period’s comments, while the flow direction of the lines illustrates the inheritance and transformation of visitor attention over time. A diachronic comparison across these three developmental stages reveals significant shifts in visitors’ perceptual priorities and emotional tone.

Phase One: Landscape Discovery and Visual Gaze Period (2010–2015). As shown on the left side of Fig 8, the thematic nodes of this phase primarily consist of concrete geographical terms such as “earth buildings,” “Tianluokeng,” “viewing platforms,” and “clusters of buildings.” The connections exhibit a relatively dispersed pattern, indicating that visitors’ perceptual logic was primarily driven by “visual curiosity” at this stage. Attention was highly concentrated on physical accessibility (e.g., “mountain roads”) and architectural uniqueness. Experiences remained unidirectional and visually fixated, lacking deep cultural emotional connections. Visitors tended to describe their experiences as “discovering a remote heritage site,” with relatively few mentions of commercial aspects.

Phase Two: Media Empowerment and Symbolic Consumption Period (2016–2019). Entering the intermediate phase, the flow of the Sankey diagram exhibited a pronounced convergence effect. With the blockbuster releases of “Cloud and Water Ballad” and “Big Fish & Begonia,” cultural symbols like “Yunshuiyao,” “film,” and “grand banyan tree” rapidly expanded, absorbing substantial traffic from the “earth-and-timber structures” node of the previous phase. This shift reflects the significant influence of film and television IP on the image of heritage sites: visitors’ focus shifted from the physical ‘architecture’ to the “scenes” romanticized by media. Frequently recurring terms in comments like “photography” and ‘recreation’ indicate that the core experience during this period centered on confirming the authenticity of cinematic symbols through photographic acts, with cultural experiences exhibiting pronounced “scenographic” characteristics.

Phase Three: Service-Driven and Experience-Driven Competition Period (2020–2024). In the evolutionary endpoint depicted on the right side of Fig 8, a critical structural divergence emerges in thematic traffic flow. While “earth buildings” and ‘architecture’ retain some attention, service and management-related nodes—such as “ticket prices,” “scenic area management,” “commercialization,” and “value for money”—have surged in prominence, becoming new focal points for traffic convergence. This clearly reflects how, in the post-pandemic era and the mature phase of mass tourism, visitors’ sensitivity thresholds have inverted: while cultural landscapes (origin) draw them in, it is service details that ultimately determine their final evaluation (destination). The thick lines flowing toward “commercialization” and “ticket prices” in the Sankey diagram vividly illustrate the intense friction tourists experience when seeking authentic experiences amid overly commercialized environments and rigid management practices.

In summary, the Sankey diagram clearly outlines the dynamic evolution of visitor perceptions: from an early “Landscape-oriented” phase, through a “Symbol-oriented” phase, ultimately evolving into a “Service-oriented” phase. This trajectory provides longitudinal evidence suggesting that, as the destination matures, visitors’ concerns increasingly extend beyond landscape appreciation to include service-related experience. In this sense, service appears to become an important component in shaping experience quality and perceptions of authenticity.

## 5. Discussion and Recommendations

This study focuses on the Tianluokeng Tulou Cluster, Yunshuiyao, Huaiyuan Lou, and Hekeng Tulou Cluster in Nanjing County, Fujian Province. Employing both quantitative text mining and qualitative interpretation of representative comments, it examines the mechanisms underlying tourist preferences, the formation of tourist-generated place image, and negative evaluations. The following sections provide detailed discussions on key research findings and theoretical contributions, along with recommendations.

### 5.1. Key Indicators Related to the Attractiveness of Traditional Villages

Research findings indicate that tourists demonstrate a significant preference for the Tianluokeng Tulou Cluster, Yunshuiyao Scenic Area, and Huaiyuan Lou, while showing relatively low interest in the Hekeng Tulou Cluster. Consequently, differences in the attractiveness of traditional villages can be discerned from visitor preferences [64].This disparity can be attributed to three core mechanisms: the symbolization of form, the scenography of emotion, and the media-enabled renarration. Among these, the role of media is particularly significant, directly influencing visitor preferences and experiences.

The Tianluokeng Tulou Cluster, with its distinctive four-round-one-square composition commonly known as four dishes and one soup, is one of the best-known components of the Fujian Tulou World Heritage property [50]. Its strong visual distinctiveness has made it one of the most recognizable examples of Fujian tulou [65]. The frequent occurrence of visitor comments like ‘If you didn’t take photos, it is like you never visited the Tulou’ is consistent with Urry’s concept of the tourist gaze, highlighting the strong role of visual spectacle in shaping tourist attention [63]. The Yunshuiyao Scenic Area achieves narrative transplantation and emotional empowerment through the film Yunshuiyao [66]. Scenes featuring century-old banyan trees, ancient paths, and waterwheels encourage visitors to recreate film scenes for photography, shifting behavior from mere sightseeing to immersive scene consumption. Sentiment analysis indicates that reviews mentioning film scenes tend to be more positive than ordinary reviews, revealing that cinematic narratives enhance visitors’ cultural identification and immersive experience [67]. However, this strong cinematic association also has side effects. Visitors show less interest in scenes unrelated to the film, such as resident areas and everyday settlement practices, which indicates that media-induced symbolism can selectively amplify some parts of place identity while obscuring others. Huaiyuan Lou has also attracted attention due to its cross-media narrative in the animated film Big Fish & Begonia [68–69]. Comments replicating scenes such as stairs and roofs indicate that film narratives may lower the threshold for understanding heritage culture, but they can also shift attention from living community practices to recognizable symbolic images.

In contrast, the low popularity of the Hekeng Tulou Cluster not only highlights challenges in tourism communication, but also shows how service affects the translation of heritage identity into tourist image. The homogenization mentioned in visitor reviews represents perceived homogenization rather than architectural reality. For ordinary visitors lacking professional expertise, insufficient guiding, weak signage, and limited storytelling make it difficult to discern subtle differences in family histories, construction techniques, clan organization, and settlement evolution among distinct tulou. Representative comments in Appendix A show this mechanism clearly: some visitors report that they mainly take photos and leave because they do not understand the wisdom behind rammed-earth construction. This case supports the cultural decoding mechanism of Culture-Service Symbiosis. Without intermediary services such as storytelling, guided interpretation, and route design, rich cultural heritage is easily simplified by visitors into repetitive visual symbols. Therefore, enhancing appeal depends not only on the landscape itself but also on translating the experience from viewing architecture to reading culture through a robust and locally grounded service system.

### 5.2. The Logical Transformation in the Formation of Traditional Village Landscapes

The empirical results of this study indicate that the service experience dimension accounts for the largest proportion (46.30%) within the overall perception structure (Table 2). This finding suggests that, in the context of Nanjing Tulou as a living heritage site, service-related factors play a prominent role in shaping visitor experience.

Under the conventional goods-dominant logic (G-D Logic) [47], traditional villages were often treated as relatively static cultural products, while services functioned mainly as logistical channels for their consumption. By contrast, the findings of this study suggest that, in the living heritage context of Nanjing Tulou, the service ecosystem has become an important medium through which visitors perceive and interpret cultural meanings. This pattern is consistent with SDL [70], but the Nanjing Tulou case further specifies how SDL works in a heritage setting: actors such as residents, guides, scenic-area managers, platform users, and tourists integrate resources through institutional arrangements including ticketing, route management, interpretation, commercial zoning, and online evaluation. Visitor value is therefore produced as value-in-use during service encounters, not only through viewing the buildings.

Consistent with the theoretical framework proposed in Section 2.5, the quantitative and semantic patterns support the Culture-Service Symbiosis framework, suggesting that service operates through two interrelated functions: cultural decoding and authenticity buffering.

First, service operates as a cultural decoder. The semantic network analysis reveals relatively strong structural connections among the nodes tour guide, explanation, and history. For many visitors without relevant architectural or cultural background knowledge, the feng shui layout, rammed-earth construction logic, clan-based social organization, and continuing resident life of the Tulou may present cognitive distance. High-quality interpretation services therefore transcend basic information delivery; they translate physical walls, courtyards, ancestral halls, and settlement layouts into understandable cultural meanings. The Hekeng case and the representative comments in Appendix A show the reverse situation: when guided interpretation is weak, visitors may describe the experience as visually repetitive and may leave after taking photographs. This mechanism explains why service is not an external supplement to culture, but part of the process through which culture becomes perceivable.

Second, service functions as an authenticity buffer. Addressing the widespread negative sentiment regarding commercialization identified in the text mining, transparent and efficient services – such as unified pricing, clear ticket rules, standardized shuttle transportation, reliable signage, and orderly commercial zoning – may reduce the perceived conflict between tourism development and cultural authenticity. The corpus shows that visitors criticize commercialization most strongly when it is combined with repeated ticket checks, unclear fees, congestion, or homogenized souvenir streets. Conversely, when commercial services are locally distinctive, clearly priced, and spatially separated from resident living areas, visitors appear more willing to accept moderate commercialization. This supports the authenticity buffering mechanism: management quality shapes whether commercial development is perceived as convenient support or as a threat to living heritage.

This interpretation offers a useful refinement to the traditional dualistic view in heritage management that tends to position tourism service development against heritage protection. From this perspective, the modernization of service infrastructure and the humanization of management practices should not be understood simply as threats to authenticity. Rather, when they are culturally sensitive and coordinated with resident life, they may constitute important conditions for sustaining the vitality of living heritage and reducing the risk of socio-cultural hollowing out. Accordingly, the destination image of traditional villages may be better understood not as a static superimposition of architectural landscapes, but as a dynamic experiential system jointly shaped by landscape features, village impressions, and service experiences.

### 5.3. Discussion of Negative Evaluation Factors in Traditional Villages

The analysis of negative perceptions indicates that visitor dissatisfaction with traditional villages is primarily associated with three factors: commercialization (42%), traffic congestion (31%), and service management issues (27%). First, commercialization poses a core conflict by eroding cultural authenticity [71]. Representative comments in Appendix A refer to additional fees for entering or climbing tulou, homogenized small commodities, and commercial streets that make different places feel similar. These comments show that visitors do not necessarily reject all tourism consumption; rather, they react negatively when consumption spaces displace local markets, resident life, or the atmosphere of the settlement. Second, traffic congestion represents a significant infrastructure constraint. Due to the mountainous setting, access to the site relies heavily on private vehicles, while parking capacity and internal transfer coordination remain limited. Non-private-vehicle visitors may encounter difficulties in accessibility, and remote Tulou clusters often require additional transportation arrangements, increasing both cost and time pressure.

Third, service management issues are closely associated with negative visitor evaluations. Problems such as repeated ticket checks, unclear fee structures, insufficient signage, limited rest areas, and uneven interpretation services may reduce overall satisfaction and weaken trust in the destination. From a broader perspective, these negative perceptions suggest the presence of an experience paradox rather than a simple rejection of tourism development. While a substantial proportion of negative evaluations focus on commercialization (42%), visitors also express expectations for modern amenities, clearer mobility arrangements, and more diversified dining options. The core issue therefore lies in whether commercial and service functions are aligned with the cultural atmosphere of the heritage setting.

When considered in relation to the findings of Section 5.2, these issues can be further understood in terms of the functioning of service-related mechanisms. In contexts where interpretive and guiding services are limited, the “cultural decoding” function of service may be weakened, resulting in relatively superficial engagement with the heritage environment. Similarly, when transportation systems, management processes, and service coordination are inefficient, the potential “buffering” role of service may be reduced, amplifying negative perceptions associated with commercialization and crowding.

Conversely, when service systems are well organized – such as through standardized transportation, transparent pricing, locally distinctive accommodation and food services, trained interpretation, and clear separation between commercial and residential areas – these mechanisms appear to function more effectively. In such cases, visitors’ tolerance toward moderate commercialization may increase, and the overall experience tends to remain more positive. These findings provide practical support for the Culture-Service Symbiosis framework and suggest that service quality plays an important role in shaping the relationship between commercialization and visitor experience in living heritage contexts.

## 6. Conclusion

This study combines longitudinal online review analysis with field-informed qualitative interpretation to examine the spatio-temporal evolution of tourists’ perceived image of Nanjing Tulou over nearly fifteen years. Based on the findings, the study summarizes its contributions in terms of theory, methodology, and practice.

### 6.1. Theoretical contributions

This study revisits the conventional goods-dominant view in heritage management, in which service is often treated primarily as logistical support, and extends the discussion of service-dominant logic (S-D Logic) within the context of living heritage tourism. By distinguishing place identity, tourist-generated place image, and place reputation, the study clarifies that online reviews do not directly represent the full identity of Nanjing Tulou or the totality of its reputation. Instead, they reveal how selected identity elements are perceived, filtered, and evaluated through visitor experience.

On this basis, the study develops a Culture-Service Symbiosis framework. Rather than viewing service as merely auxiliary to heritage consumption, this framework suggests that service plays two interrelated interpretive functions in living heritage destinations. First, as a cultural decoder, high-quality guided tours, signage, digital tools, and storytelling services may help reduce cognitive friction for visitors by translating relatively static architectural symbols into more accessible cultural meanings. Second, as an authenticity buffer, transparent pricing, coherent transport management, and culturally sensitive commercial regulation may help alleviate negative reactions associated with commercialization, thereby supporting visitors’ perceptions of everyday cultural authenticity.

Taken together, this framework provides a useful perspective for understanding how commercialization and perceived authenticity may coexist in living heritage destinations. More broadly, it contributes to heritage tourism research by highlighting that service is not only an operational condition of visitation, but also an important component in the production, interpretation, and stabilization of heritage experience. In this sense, the study highlights the need to understand heritage not only as an object of visual consumption, but also as an experience mediated through service-related interpretation and management.

### 6.2. Methodological contributions

To address the limitations of existing studies that rely primarily on cross-sectional data and pay limited attention to long-term change, this study adopts an analytical strategy that combines stage-based temporal topic modeling with Sankey-based topic-evolution analysis. Drawing on nearly fifteen years of longitudinal review data, the analysis identifies an observable shift in visitor attention, from an earlier emphasis on visual appreciation, to media-related consumption, and later to stronger concern with service-related experience. This approach helps visualize thematic trajectories within large-scale textual data and demonstrates the value of incorporating temporal dimensions into heritage tourism research. In this sense, the study provides a useful methodological reference for examining the dynamic evolution of visitor perception in heritage destinations.

### 6.3. Practical implications

Based on the analysis of negative feedback and the structural dimensions of visitor perception, this study translates the Culture-Service Symbiosis framework into evidence-based management recommendations for Nanjing Tulou and similar living heritage sites. First, in response to complaints concerning repeated ticket checks, unclear fees, and fragmented management, managers may establish a unified ticketing and verification system across village clusters, publish transparent fee lists at entrances and digital platforms, and use time-slot or visitor-flow management during peak periods. These measures are feasible because they mainly require coordination among existing scenic-area operators and can reduce administrative friction without changing the heritage fabric.

Second, to address the concern that over-commercialization may erode cultural authenticity, managers should implement clearer commercial zoning and culturally sensitive stall regulation. Homogenized souvenir stalls should be kept away from core residential courtyards and ritual or clan spaces, while locally grounded food, craft, agricultural products, and resident-led interpretation may be prioritized. This approach protects resident everyday life and community agency while allowing moderate commercial activity to support local livelihoods.

Third, the recurring complaints concerning accessibility point to targeted infrastructural improvement. Given the high proportion of self-driving regional tourists and the frequent mention of traffic congestion in the review data, management may optimize external parking guidance, shuttle frequency, internal transfer signage, and route information for non-self-driving visitors. These changes would directly address the traffic and parking problems identified in the corpus while avoiding large-scale construction that could damage the mountain settlement landscape.

Finally, building on the strong visitor appeal associated with cinematic and television IPs, management may further improve cultural interpretation strategies. Trained guides, multilingual signage, short digital interpretation modules, and carefully designed VR/AR content can connect film-related symbols with architecture, settlement history, resident life, and clan organization. Such an approach would strengthen the destination’s cultural decoding function and help shift visitor behavior from primarily visual and photo-oriented consumption toward deeper cultural engagement.

### 6.4. Limitations and future research

However, this study also has certain limitations. The main data sources primarily focused on Ctrip and Dianping and therefore mainly reflect the tourist-generated place image formed on major Chinese travel platforms. To contextualize this limitation, this study conducted a supplementary reading of publicly visible English-language reviews on international-facing platforms such as Tripadvisor and Trip.com [60–61]. This contextual check suggested that international-facing platform content also mentions architectural uniqueness, World Heritage value, transport difficulty, interpretation, and commercialization. Nevertheless, because these pages mix international tourists, domestic users, platform-generated travel notes, and translated content, and because they lack comparable full-period metadata, they were not merged into the main statistical corpus. Therefore, the conclusions should still be understood as primarily based on Chinese-platform review data. Future research should collect multilingual data from TripAdvisor, Instagram, Google Maps, Xiaohongshu, Weibo, Douyin, and other platforms, and should incorporate image and video content such as vlogs to make the conclusions more multidimensional and comprehensive. In addition, online reviewers are more likely to be younger or middle-aged internet-active travelers, and therefore may not fully represent the perceptions of older tourists or visitors who are less inclined to share their experiences online.

Despite these limitations, the study suggests that systematic analysis of visitor reviews can offer useful insights for tourism destination management. A closer understanding of visitors’ concerns and expectations may help managers identify areas for improving facilities, interpretation, and service quality. In this respect, online reviews can be understood not only as indicators of destination reputation, but also as a source of diagnostic information about emerging management issues. Negative reviews, in particular, may help identify recurring problems that deserve attention and may support more responsive destination planning and service adjustment.

## Supporting information

S1 File Appendix A. (DOCX)
