# Supplementary material for: Tourist perceptions and living heritage experiences in Nanjing Tulou traditional villages, Fujian, China: A social media data analysis
Source: PLoS One. 2026 Aug 6;21(8):e0354702. doi: 10.1371/journal.pone.0354702 (PMC13446676; doi:10.1371/journal.pone.0354702)
Supplement: Supplementary file 1 [file pone.0354702.s001.docx]

Appenidx A:

| Primary Code | Secondary code | Original text |
| --- | --- | --- |
| Architectural Heritage | Tulou construction techniques, feng shui layout, and historical origins | “The ‘four dishes and one soup’ tulous cluster like growth rings emerging from the earth, their rammed-earth walls permeated with the deep fragrance of time”.  “Built according to the generative sequence of the Five Elements—Metal, Wood, Water, Fire, and Earth—it is hailed as a marvel of world architecture”.  “Hegui Lou stands upon marshland, where a light step can feel the ground tremble—a Noah’s Ark upon dry land”. |
| Cinematic Cultural Landscape | Film location symbols, scene recreation, emotional resonance | “Huaiyuan Lou is the home of Qiu from Big Fish & Begonia. Under the starlit night, it feels like stepping into the world of animation”.  “The banyan tree beside the large waterwheel witnessed the heartbreaking farewell scene in the film ‘Yunshuiyao’,where love was whispered in the wind amidst the drizzle”.  “Changjiao Village was renamed ‘Yunshuiyao’ after the film, and its poetic name makes the blue skies and clear waters even more alluring”. |
| Landscape and Pastoral | Landscape scenery, ancient tree ecology, pastoral poetry | “Thirteen century-old banyan trees stand guard over the village, the largest ancient banyan requiring ten people to encircle its trunk, its canopy shading the sun like an umbrella”.  “Green stone slabs wind through terraced fields, Emerald tea mountains trace the earth’s fingerprints; Sunset smoke drifts over rammed-earth eaves”.  “Along the ancient streamside path, the gentle murmur of flowing water can be heard; As the waterwheel turns, one feels transported to a hidden paradise”. |
| Commercialized nostalgia | Commercialization erodes culture, consumer traps, loss of authenticity | “Admission is 90 yuan, with additional fees for climbing the earthen buildings. The prevalence of Yiwu small commodities diminishes the cultural ambiance”.  “Compared to the simplicity of Tianluokeng, Yunshuiyao is overrun with tourists and heavily commercialized”.  “The night view of the lantern-lit earthen buildings is beautiful, but the homogenized shops make it feel like ‘everywhere looks the same’”. |
| Hakka Rural Life | Hakka Folk Customs, Market Life, Culinary Traditions | “In the courtyard’s play of light and shadow, one seems to hear the echoes of Hakka folk gathered around the stove, sharing stories late into the night”.  “Dawn tulou market: Villagers sell wild tea berries, pickled noodles, and pork knuckle noodles, brimming with the warmth of everyday life”.  “Must-try four delicacies: salted duck, stir-fried bamboo shoots with pork, small river shrimp, and golden thread orchid tea”. |
| Experience Threshold | Transportation shortcomings, inadequate facilities, and superficial experiences | “The remote location is ideal for self-driving tourists, but non-self-driving visitors face the dilemma of being unable to enter or leave”.  “Primarily stone paths, so flat shoes are recommended; Lack of rest areas leads to fatigue”.  “Visitors linger to take photos but lack deeper cultural engagement; the absence of guided tours prevents them from appreciating the wisdom of rammed earth construction”. |
