# Supplementary material for: Tourist perceptions and living heritage experiences in Nanjing Tulou traditional villages, Fujian, China: A social media data analysis
Source: PLoS One. 2026 Aug 6;21(8):e0354702. doi: 10.1371/journal.pone.0354702 (PMC13446676; doi:10.1371/journal.pone.0354702)
Supplement: Supplementary file 2 [file pone.0354702.s005.docx]

Response to Reviewers

Manuscript ID: PONE-D-26-04498R1

Title: Tourist Perceptions and Living Heritage Experiences in Nanjing Tulou Traditional Villages, Fujian, China: A Social Media Data Analysis

Journal: PLOS ONE

Dear Academic Editor and Reviewers, thank you for the constructive comments on our revised manuscript. We have prepared a further revision that strengthens the theoretical framework, clarifies the research object, adds more qualitative interpretation of the review evidence, corrects World Heritage wording, and more explicitly acknowledges the representativeness limits of the dataset. All changes are highlighted in the marked manuscript.

Response to Reviewer #1

Comment 1: The overall innovation is insufficient and the manuscript relies too heavily on textual statistics.

Response: We revised the manuscript to make the original contribution clearer and less dependent on descriptive statistics alone. The revised theoretical framework now distinguishes place identity, place image, and place reputation, and defines Culture-Service Symbiosis before the empirical sections. In the Discussion, the statistical results are interpreted with concrete comment-based cases such as Hekeng's perceived homogenization, inadequate interpretation, repeated ticket checks, transport friction, and commercialization. These changes appear in Sections 2.4, 2.5, 5.1, 5.2, and 5.3, and clarify that the paper's contribution is not only the frequency distribution of review terms, but the explanation of how service mediates the transformation from living heritage identity into tourist-generated place image. Manuscript location: Sections 2.4-2.5, lines 205-280; Sections 5.1-5.3, lines 1101-1227.

Comment 2: The analysis remains shallow; why service is important should be further analyzed qualitatively with practical cases.

Response: We added qualitative interpretation throughout the Discussion. The revised manuscript explains the Hekeng case as a cultural decoding failure: without guides, storytelling, signage, and route design, visitors tend to reduce distinct tulou histories and construction techniques to repetitive visual symbols. We also explain authenticity buffering through specific negative-review themes, including repeated ticket checks, unclear fees, congestion, and homogenized souvenir streets. These cases, now discussed mainly in Sections 5.1-5.3 and Appendix A, connect the text-mining results with the mechanisms behind service importance. Manuscript location: Sections 5.1-5.3, lines 1101-1227; Appendix A, lines 1317-1417.

Comment 3: The Culture-Service Symbiosis concept is theoretically hollow and requires rigorous support.

Response: We have moved the Culture-Service Symbiosis framework into the theoretical framework section and defined it explicitly. The revised framework is derived from SDL, authenticity theory, living heritage theory, and the place identity/image/reputation distinction. It identifies two mechanisms: cultural decoding, through which interpretation and guidance help visitors understand Tulou architecture and resident life; and authenticity buffering, through which transparent and culturally sensitive management reduces the negative effects of commercialization and service friction on perceived authenticity. These changes are made in Section 2.5 and further supported in Sections 5.2 and 6.1. Manuscript location: Section 2.5, lines 231-280; Section 5.2, lines 1142-1190; Section 6.1, lines 1233-1255.

Comment 4: Practical recommendations are too abstract and should be tied to the results and feasibility.

Response: We revised the practical implications to be more operational. The revised recommendations include unified ticketing and verification, transparent fee lists, time-slot and visitor-flow management, commercial zoning that protects residential and ritual spaces, locally grounded products and resident-led interpretation, shuttle and parking coordination, multilingual signage, trained guides, and short digital interpretation modules. These revisions appear in Section 6.3, and each recommendation is tied to a recurring issue identified in the corpus. Manuscript location: Section 6.3, lines 1266-1292.

Comment 5: The data are limited to domestic tourism platforms and lack international comparison.

Response: We added a supplementary international-facing platform triangulation step. Publicly visible English-language reviews on platforms such as Tripadvisor and Trip.com were read as contextual evidence rather than incorporated into the main quantitative corpus. The revision explains that this international-facing platform content also mentions architectural uniqueness, World Heritage value, transport difficulty, interpretation, and commercialization. It also clarifies why these data were not merged into the main statistical corpus: the pages mix international tourists, domestic users, platform-generated travel notes, and translated content, and they do not provide comparable full-period metadata. We therefore keep the main corpus unchanged while explicitly limiting the claims to Chinese-platform tourist-generated place image and identifying systematic multilingual comparison as a future research direction. These changes appear in Sections 3.4 and 6.4. Manuscript location: Section 3.4, lines 395-433; Section 6.4, lines 1293-1316.

Response to Reviewer #3

Comment 1: Add the distinction among place identity, place image, and place reputation.

Response: We added a new subsection, 'Place Identity, Place Image, and Place Reputation,' citing Matlovičová (2024) and Michalková (2014). The revised manuscript clarifies that the place identity of the wider Fujian Tulou heritage landscape includes rammed-earth architecture, settlement morphology, clan organization, resident everyday life, ritual and customary practices, mountain landscape settings, and World Heritage status. The study measures tourist-generated place image reconstructed from online review texts, not the full identity or total reputation of the destination. This revision appears in Section 2.4. Manuscript location: Section 2.4, lines 205-230.

Comment 2: Define Culture-Service Symbiosis earlier and connect it to the place image framework.

Response: We now define Culture-Service Symbiosis in the theoretical framework rather than only in the Discussion and Conclusion. The revised framework treats service as a mediating interface between place identity and tourist-generated place image. Good interpretation, transparent management, and culturally sensitive services help visitors decode heritage identity into richer place image, while poor service and excessive commercialization may distort this transformation and create reputational risk. This revision appears in Section 2.5 and is applied in Sections 5.2 and 6.1. Manuscript location: Section 2.5, lines 231-280; Section 5.2, lines 1142-1190; Section 6.1, lines 1233-1255.

Comment 3: Derive the mechanisms from SDL, authenticity theory, and living heritage theory, rather than simply saying service matters.

Response: We revised the SDL discussion to use core concepts including value co-creation, resource integration, service ecosystems, actors, institutional arrangements, and value-in-use. In the revised manuscript, residents, guides, managers, platform users, and tourists are treated as actors who integrate resources through ticketing, route management, interpretation, commercial zoning, and online evaluation. This provides the theoretical basis for the two mechanisms of cultural decoding and authenticity buffering. These revisions appear in Sections 2.5 and 5.2. Manuscript location: Section 2.5, lines 231-280; Section 5.2, lines 1142-1190.

Comment 4: Correct World Heritage wording, verify the UNESCO phrase, and nuance Hakka culture.

Response: We corrected the manuscript to state that 'Fujian Tulou' is the UNESCO World Heritage property and that the Nanjing Tulou Scenic Area is a component of that wider property. We removed the unverified wording that UNESCO described Tianluokeng as 'the pinnacle of geometric aesthetics in rammed-earth architecture' and replaced it with a more cautious description of Tianluokeng's distinctive four-round-one-square composition. We also revised essentializing references to Hakka culture by adding resident everyday life, clan organization, ritual and customary practices, foodways, market scenes, and community agency. These changes appear in Sections 3.1, 5.1, and 6.3. Manuscript location: Section 3.1, lines 282-316; Section 5.1, lines 1101-1141; Section 6.3, lines 1266-1292.

Additional language and clarity revisions

Response: We revised terminology throughout the manuscript to distinguish tourist perception, tourist-generated place image, destination reputation, cultural authenticity, and living heritage identity. We also made the limitations section more precise and clarified that the international-facing review scan is supplementary rather than part of the main statistical corpus. Manuscript location: Sections 2.4-2.5, lines 205-280; Section 3.4, lines 395-433; Sections 5.1-5.3, lines 1101-1227; Section 6.4, lines 1293-1316.

Sincerely,

The Authors
