# Supplementary material for: Tourist perceptions and living heritage experiences in Nanjing Tulou traditional villages, Fujian, China: A social media data analysis
Source: PLoS One. 2026 Aug 6;21(8):e0354702. doi: 10.1371/journal.pone.0354702 (PMC13446676; doi:10.1371/journal.pone.0354702)
Supplement: Supplementary file 3 [file pone.0354702.s002.doc]

Overall, this article has certain theoretical and practical value. It focuses on the research topic of tourist perception and living heritage experience in the traditional villages of Tulou in Nanjing, Fujian Province, closely addressing the core issue of balancing cultural heritage protection and tourism experience. It aligns with the current research hotspots on the live inheritance and tourism development of traditional villages. The research results have certain reference significance for optimizing tourism services and protecting the living heritage of similar heritage sites. However, from the perspective of research methods, the Python data scraping and ROSTCM6.0 text analysis (frequent words, sentiment analysis, DTM, etc.) used in the article are all relatively common and mature methods in current tourism perception research. The research design and analysis approach lack obvious innovative breakthroughs. They did not develop or improve characteristic research methods based on the particularity of the research topic (such as Hakka culture of Tulou and the characteristics of living heritage), nor did they form a unique perspective in the integration and application of existing methods. To some extent, this affected the academic contribution and competitiveness of the research.

1. Strengthen the specificity of the case studies: Add specific samples of Nanjing Tulou (such as Tianluogeng Tulou Group, Heikeng Tulou Group, etc.), clarify the basis for case selection (such as popularity, tourism development degree, and the preservation status of the living heritage), and enhance the representativeness and rigor of the research cases.

2. Refine the data collection methods and improve data transparency and reproducibility: The summary only mentions "collecting tourist review content from Ctrip and Dianping over the past 15 years using Python 3.12.3", further details are needed: specify the exact time range of data collection (such as 2009-2024), the sample selection criteria (such as the definition of valid reviews: excluding advertisements, duplicates, and those without substantive content), the sample size, the ethical norms for data scraping (such as whether platform authorization was obtained, whether user information was anonymized), and the core code logic of Python scraping (which can be uploaded as supplementary materials), meeting the core requirements of PLOS ONE for the reproducibility of research methods.

3. Supplement the rationality demonstration of the research methods, strengthen the adaptability of the methods, and improve the visual presentation of the theoretical framework: Clearly explain why the Service-Dominant Logic (SDL) framework was chosen as the research perspective, and elaborate on the adaptability of this framework to the "tourist perception, living heritage experience" and "culture-service symbiosis" model - such as the core viewpoint of SDL framework that "service is the core of value co-creation", how it supports the research core of "service as the medium for the transmission of living heritage culture", and how this framework provides theoretical support for the division of the tourist perception dimension and the analysis of the experience mechanism. At the same time, explain the advantages of combining ROSTCM6.0 software with Python (such as Python's efficient data scraping and preprocessing, ROSTCM6.0's precise text mining and sentiment analysis, and the collaborative improvement of research efficiency and analysis accuracy), as well as the division and correlation of the four methods: frequency word analysis, network semantic analysis, sentiment analysis, and dynamic topic modeling (DTM), avoiding method overloading, and clarifying the supporting role of each method for the research objectives (such as frequency word analysis identifying core perception points, network semantic analysis mining the correlation of perception dimensions, sentiment analysis quantifying tourist attitudes, and DTM tracking the dynamic changes in perception). Enhance the rigor of the research methods. Additionally, in the theoretical framework section, it is recommended to present the research logic of the paper in a clear graphical form, drawing a research framework diagram to visually display the complete logical chain of "theoretical basis (SDL framework) - research methods (multi-text analysis methods) - research content (tourist perception dimensions, sentiment tendencies, theme changes) - research conclusion (culture-service symbiosis model)", marking the relationships between each link, making the research logic clearer and more understandable.

4. Strengthen the depth of result analysis, supplement the analysis of differences and mechanisms: The existing results only present the dimensions of tourist perception, sentiment tendencies, and theme changes. Further details are needed for the analysis of differences: such as the differences in perception among tourists of different age groups and different tourist sources, the driving mechanisms of changes in tourist perception in different tourism development stages (such as policy guidance, media promotion, and infrastructure improvement). At the same time, for the core result of "service experience taking the leading position", supplement specific cases (such as specific problems in transportation and cost management or quality service cases), to enhance the persuasiveness of the results. 5. Improve the analysis of negative evaluations and enhance the practical value of the research: The abstract only mentions three sources of negative evaluations (commercialization eroding cultural authenticity, insufficient transportation accessibility, and service management deficiencies), and further detailed analysis is needed: The specific manifestations of each negative evaluation (such as the specific forms of commercialization: over-commercialized stores, false cultural experiences, etc.; specific scenarios of insufficient transportation accessibility: external transportation outside the scenic area, internal connections, etc.), as well as the ranking of the influence degree of different negative factors, will provide support for proposing targeted improvement measures in the future.

6. Strengthen the innovation and rationality demonstration of the "culture-service symbiosis" model: The "culture-service symbiosis" model proposed in the abstract is the core innovation point, and it is necessary to further supplement the specific connotation, constituent elements (such as specific indicators at the cultural level and service level), and operation mechanism of the model, clarify the differences and connections between this model and existing related theories (such as the SDL framework, cultural heritage protection theory), and demonstrate the innovation and feasibility of the model; at the same time, supplement the verification methods of the model (such as combining case data, interview data, etc.), to avoid the model being proposed too abruptly and enhance the theoretical contribution of the research.

7. The clarity of the 293-row location map needs to be improved, and the text on the map cannot be clearly seen.
