# Supplementary material for: Tourist perceptions and living heritage experiences in Nanjing Tulou traditional villages, Fujian, China: A social media data analysis
Source: PLoS One. 2026 Aug 6;21(8):e0354702. doi: 10.1371/journal.pone.0354702 (PMC13446676; doi:10.1371/journal.pone.0354702)
Supplement: Peer review history

# Peer review: Decision Letter 0

**DOI:** 10.1371/journal.pone.0354702.r001

**Authors:** Yile Chen

2 Mar 2026

PONE-D-26-04498

Tourist Perceptions and Living Heritage Experiences in Fujian Nanjing Tulou Traditional Villages Based on Social Media Data Analysis

PLOS One

Dear Dr. Wang,

Thank you for submitting your manuscript to PLOS ONE. After careful consideration, we feel that it has merit but does not fully meet PLOS ONE’s publication criteria as it currently stands. Therefore, we invite you to submit a revised version of the manuscript that addresses the points raised during the review process.

Please submit your revised manuscript by  Apr 16 2026 11:59PM. If you will need more time than this to complete your revisions, please reply to this message or contact the journal office at plosone@plos.org. When you're ready to submit your revision, log on to https://www.editorialmanager.com/pone/ and select the 'Submissions Needing Revision' folder to locate your manuscript file.

Please include the following items when submitting your revised manuscript:

- A letter that responds to each point raised by the academic editor and reviewer(s). You should upload this letter as a separate file labeled 'Response to Reviewers'.
- A marked-up copy of your manuscript that highlights changes made to the original version. You should upload this as a separate file labeled 'Revised Manuscript with Track Changes'.
- An unmarked version of your revised paper without tracked changes. You should upload this as a separate file labeled 'Manuscript'.

If you would like to make changes to your financial disclosure, please include your updated statement in your cover letter. Guidelines for resubmitting your figure files are available below the reviewer comments at the end of this letter.

If applicable, we recommend that you deposit your laboratory protocols in protocols.io to enhance the reproducibility of your results. Protocols.io assigns your protocol its own identifier (DOI) so that it can be cited independently in the future. For instructions see: https://journals.plos.org/plosone/s/submission-guidelines#loc-laboratory-protocols. Additionally, PLOS ONE offers an option for publishing peer-reviewed Lab Protocol articles, which describe protocols hosted on protocols.io. Read more information on sharing protocols at https://plos.org/protocols?utm_medium=editorial-email&utm_source=authorletters&utm_campaign=protocols.

We look forward to receiving your revised manuscript.

Kind regards,

Yile Chen, Ph.D. in Architecture

Academic Editor

PLOS One

Journal Requirements:

When submitting your revision, we need you to address these additional requirements.

1. Please ensure that your manuscript meets PLOS ONE's style requirements, including those for file naming. The PLOS ONE style templates can be found at

https://journals.plos.org/plosone/s/file?id=wjVg/PLOSOne_formatting_sample_main_body.pdf and

https://journals.plos.org/plosone/s/file?id=ba62/PLOSOne_formatting_sample_title_authors_affiliations.pdf

2. Please note that PLOS One has specific guidelines on code sharing for submissions in which author-generated code underpins the findings in the manuscript. In these cases, we expect all author-generated code to be made available without restrictions upon publication of the work. Please review our guidelines at https://journals.plos.org/plosone/s/materials-and-software-sharing#loc-sharing-code and ensure that your code is shared in a way that follows best practice and facilitates reproducibility and reuse.

3. In your Methods section, please include additional information about your dataset and ensure that you have included a statement specifying whether the collection and analysis method complied with the terms and conditions for the source of the data.

4. We note that the grant information you provided in the ‘Funding Information’ and ‘Financial Disclosure’ sections do not match.

When you resubmit, please ensure that you provide the correct grant numbers for the awards you received for your study in the ‘Funding Information’ section.

5. Thank you for stating the following financial disclosure:

“2022 National Social Science Fund Art Project "Research on Modern Jiangnan Urban Park Design" (Project No. 22BG124); 2022 National Social Science Foundation Art Major Project “Research on Chinese Urban Image Design” (Project No. 22ZD18).”

Please state what role the funders took in the study. If the funders had no role, please state: "The funders had no role in study design, data collection and analysis, decision to publish, or preparation of the manuscript."

If this statement is not correct you must amend it as needed.

Please include this amended Role of Funder statement in your cover letter; we will change the online submission form on your behalf.

6. Thank you for stating the following in the Acknowledgments Section of your manuscript:

“Education and Research Project for Middle-aged and Young Teachers in Fujian Province (Social Science Category): Digital Protection and Revitalization Research of Ancient Villages in Minxi Area (JAS20352)

2.Fujian Social Science Fund Project: Research on the Cultural Activation Mechanism of Hakka Traditional Villages in Western Fujian from the Perspective of Rural Revitalization (FJ2024X008)”

We note that you have provided funding information that is currently declared in your Funding Statement. However, funding information should not appear in the Acknowledgments section or other areas of your manuscript. We will only publish funding information present in the Funding Statement section of the online submission form.

Please remove any funding-related text from the manuscript and let us know how you would like to update your Funding Statement. Currently, your Funding Statement reads as follows:

“2022 National Social Science Fund Art Project "Research on Modern Jiangnan Urban Park Design" (Project No. 22BG124); 2022 National Social Science Foundation Art Major Project “Research on Chinese Urban Image Design” (Project No. 22ZD18).”

Please include your amended statements within your cover letter; we will change the online submission form on your behalf.

7. PLOS requires an ORCID iD for the corresponding author in Editorial Manager on papers submitted after December 6th, 2016. Please ensure that you have an ORCID iD and that it is validated in Editorial Manager. To do this, go to ‘Update my Information’ (in the upper left-hand corner of the main menu), and click on the Fetch/Validate link next to the ORCID field. This will take you to the ORCID site and allow you to create a new iD or authenticate a pre-existing iD in Editorial Manager.

8. Your ethics statement should only appear in the Methods section of your manuscript. If your ethics statement is written in any section besides the Methods, please move it to the Methods section and delete it from any other section. Please ensure that your ethics statement is included in your manuscript, as the ethics statement entered into the online submission form will not be published alongside your manuscript.

9. We note that Figure 1 in your submission contain copyrighted images. All PLOS content is published under the Creative Commons Attribution License (CC BY 4.0), which means that the manuscript, images, and Supporting Information files will be freely available online, and any third party is permitted to access, download, copy, distribute, and use these materials in any way, even commercially, with proper attribution. For more information, see our copyright guidelines: http://journals.plos.org/plosone/s/licenses-and-copyright.

We require you to either (1) present written permission from the copyright holder to publish these figures specifically under the CC BY 4.0 license, or (2) remove the figures from your submission:

a. You may seek permission from the original copyright holder of Figure 1 to publish the content specifically under the CC BY 4.0 license.

We recommend that you contact the original copyright holder with the Content Permission Form (http://journals.plos.org/plosone/s/file?id=7c09/content-permission-form.pdf) and the following text:

“I request permission for the open-access journal PLOS ONE to publish XXX under the Creative Commons Attribution License (CCAL) CC BY 4.0 (http://creativecommons.org/licenses/by/4.0/). Please be aware that this license allows unrestricted use and distribution, even commercially, by third parties. Please reply and provide explicit written permission to publish XXX under a CC BY license and complete the attached form.”

Please upload the completed Content Permission Form or other proof of granted permissions as an "Other" file with your submission.

In the figure caption of the copyrighted figure, please include the following text: “Reprinted from [ref] under a CC BY license, with permission from [name of publisher], original copyright [original copyright year].”

b. If you are unable to obtain permission from the original copyright holder to publish these figures under the CC BY 4.0 license or if the copyright holder’s requirements are incompatible with the CC BY 4.0 license, please either i) remove the figure or ii) supply a replacement figure that complies with the CC BY 4.0 license. Please check copyright information on all replacement figures and update the figure caption with source information. If applicable, please specify in the figure caption text when a figure is similar but not identical to the original image and is therefore for illustrative purposes only.

10. We note that Figure 1 in your submission contain map/satellite images which may be copyrighted. All PLOS content is published under the Creative Commons Attribution License (CC BY 4.0), which means that the manuscript, images, and Supporting Information files will be freely available online, and any third party is permitted to access, download, copy, distribute, and use these materials in any way, even commercially, with proper attribution. For these reasons, we cannot publish previously copyrighted maps or satellite images created using proprietary data, such as Google software (Google Maps, Street View, and Earth). For more information, see our copyright guidelines: http://journals.plos.org/plosone/s/licenses-and-copyright.

We require you to either (1) present written permission from the copyright holder to publish these figures specifically under the CC BY 4.0 license, or (2) remove the figures from your submission:

a. You may seek permission from the original copyright holder of Figures 1 to publish the content specifically under the CC BY 4.0 license.

We recommend that you contact the original copyright holder with the Content Permission Form (http://journals.plos.org/plosone/s/file?id=7c09/content-permission-form.pdf) and the following text:

“I request permission for the open-access journal PLOS ONE to publish XXX under the Creative Commons Attribution License (CCAL) CC BY 4.0 (http://creativecommons.org/licenses/by/4.0/). Please be aware that this license allows unrestricted use and distribution, even commercially, by third parties. Please reply and provide explicit written permission to publish XXX under a CC BY license and complete the attached form.”

Please upload the completed Content Permission Form or other proof of granted permissions as an "Other" file with your submission.

In the figure caption of the copyrighted figure, please include the following text: “Reprinted from [ref] under a CC BY license, with permission from [name of publisher], original copyright [original copyright year].”

b. If you are unable to obtain permission from the original copyright holder to publish these figures under the CC BY 4.0 license or if the copyright holder’s requirements are incompatible with the CC BY 4.0 license, please either i) remove the figure or ii) supply a replacement figure that complies with the CC BY 4.0 license. Please check copyright information on all replacement figures and update the figure caption with source information. If applicable, please specify in the figure caption text when a figure is similar but not identical to the original image and is therefore for illustrative purposes only.

The following resources for replacing copyrighted map figures may be helpful:

USGS National Map Viewer (public domain): http://viewer.nationalmap.gov/viewer/

The Gateway to Astronaut Photography of Earth (public domain): http://eol.jsc.nasa.gov/sseop/clickmap/

Maps at the CIA (public domain): https://www.cia.gov/library/publications/the-world-factbook/index.html and https://www.cia.gov/library/publications/cia-maps-publications/index.html

NASA Earth Observatory (public domain): http://earthobservatory.nasa.gov/

Landsat: http://landsat.visibleearth.nasa.gov/

USGS EROS (Earth Resources Observatory and Science (EROS) Center) (public domain): http://eros.usgs.gov/#

Natural Earth (public domain): http://www.naturalearthdata.com/

11. Thank you for uploading your study's underlying data set. Unfortunately, the repository you have noted in your Data Availability statement does not qualify as an acceptable data repository according to PLOS's standards.

At this time, please upload the minimal data set necessary to replicate your study's findings to a stable, public repository (such as figshare or Dryad) and provide us with the relevant URLs, DOIs, or accession numbers that may be used to access these data. For a list of recommended repositories and additional information on PLOS standards for data deposition, please see https://journals.plos.org/plosone/s/recommended-repositories.

12. If the reviewer comments include a recommendation to cite specific previously published works, please review and evaluate these publications to determine whether they are relevant and should be cited. There is no requirement to cite these works unless the editor has indicated otherwise.

Additional Editor Comments:

The author needs to further clarify the methodology and data situation for social media data analysis; the typical representativeness of Fujian Tulou; and the replicability of the analytical framework. A review of similar literature from recent years could be added. Other aspects can be referenced from the reviewers' comments.

[Note: HTML markup is below. Please do not edit.]

Reviewers' comments:

Reviewer's Responses to Questions

**Comments to the Author**

1. Is the manuscript technically sound, and do the data support the conclusions?

The manuscript must describe a technically sound piece of scientific research with data that supports the conclusions. Experiments must have been conducted rigorously, with appropriate controls, replication, and sample sizes. The conclusions must be drawn appropriately based on the data presented.

Reviewer #1: Yes

Reviewer #2: No

**********

2. Has the statistical analysis been performed appropriately and rigorously?

Reviewer #1: Yes

Reviewer #2: Yes

**********

3. Have the authors made all data underlying the findings in their manuscript fully available?

The PLOS Data policy requires authors to make all data underlying the findings described in their manuscript fully available without restriction, with rare exception (please refer to the Data Availability Statement in the manuscript PDF file). The data should be provided as part of the manuscript or its supporting information, or deposited to a public repository. For example, in addition to summary statistics, the data points behind means, medians and variance measures should be available. If there are restrictions on publicly sharing data—e.g. participant privacy or use of data from a third party—those must be specified.

Reviewer #1: Yes

Reviewer #2: Yes

**********

4. Is the manuscript presented in an intelligible fashion and written in standard English?

PLOS ONE does not copyedit accepted manuscripts, so the language in submitted articles must be clear, correct, and unambiguous. Any typographical or grammatical errors should be corrected at revision, so please note any specific errors here.

Reviewer #1: Yes

Reviewer #2: No

**********

5. Review Comments to the Author

Please use the space provided to explain your answers to the questions above. You may also include additional comments for the author, including concerns about dual publication, research ethics, or publication ethics. (Please upload your review as an attachment if it exceeds 20,000 characters)

Reviewer #1: Overall Comment�The research methods adopted in this paper are relatively rigorous, and the research discussion is closely aligned with the theme of tourist perception and living heritage experience in traditional villages. The overall logical flow of the paper is progressive, with a complete research framework that holds certain academic value. However, the paper still has shortcomings in polishing details, excavating content, and expressing research methods, which need further revision and improvement.

1. The author's affiliated institution should supplement the annotation of the secondary unit.

2. Avoid expressions such as "filling the gap in..." in the introduction; alternative phrasings like "enhancing the research depth of..." are recommended.

3. The literature review lays a theoretical foundation for empirical research, yet it lacks a thorough review of exclusive studies on the core concept of "living heritage", resulting in insufficient alignment with the research theme. In addition, some viewpoints are repetitive and can be appropriately merged and integrated.

4. The specific source of the architectural photograph in Figure 1 should be indicated.

5. The research methods in Sections 3.2, 3.3 and 3.4 are commonly used; the wording should be optimized in combination with the research content to reduce the repetition rate with similar literature.

6. Table 5 can be adjusted to a dendrogram showing the hierarchical relationships of first-level, second-level and other codings, with the detailed characteristic summary table placed in the appendix.

7. In Chapter 3 (Data and Methods), the data source is single and the deviation analysis is superficial; the division of research stages can be carried out in conjunction with the key events of the actual development of the scenic area. Moreover, the key parameters of dynamic topic modeling are not disclosed, and the dimension of reliability verification is single.

8. For Chapters 4 and 5, the integration of quantitative data with analysis and discussion should be strengthened. Core quantitative indicators need to be closely embedded in the entire process of perception interpretation and theoretical deduction, with quantitative evidence matched to practical recommendations. Data should serve as the core support for the research content to achieve an organic connection between data, theory and practice.

Reviewer #2: 1. Regarding research ethics: The core data of the article is sourced from tourist reviews on travel platforms such as Ctrip and Dianping. Such data belong to publicly shared content by users, but still need to strictly follow research ethics guidelines. It is recommended that the author clarify the ethical compliance of data scraping during the revision process - whether official authorization from the relevant platforms has been obtained, and whether personal information of tourists (such as nicknames, avatars, geographical locations, etc.) has been thoroughly de-identified to avoid privacy leakage; at the same time, supplement an ethical statement on data usage, clearly stating that the data is only used for academic research and not for commercial purposes, which is in line with the strict requirements of PLOS ONE for research ethics, and avoiding issues related to ethics affecting the review of the manuscript.

2. Regarding dual publication and academic integrity: Please confirm that the research content, data, and conclusions of this article have not been published in other journals (including Chinese and English core journals, conference proceedings), nor have they been simultaneously submitted to other journals. There is no situation of dual publication or multiple submissions of the same manuscript; at the same time, clearly state that all references, data, and methods in the article have been properly labeled, and there is no plagiarism, fraud, data forgery, or data tampering, etc. in academic misconduct. It is recommended to add an author's integrity statement in the manuscript to ensure compliance with publication ethics requirements.

3. Other supplementary suggestions: When revising the manuscript, the author should pay attention to the objectivity and neutrality of the research. Especially when analyzing negative issues such as "commercialization eroding cultural authenticity", one should avoid subjective assumptions and conduct rational analysis by combining specific data and cases. At the same time, enhance the completeness of the literature review, summarize the research progress and shortcomings of current similar studies, clarify the research positioning and marginal contributions of this paper, and further highlight the academic value of the research. Additionally, it is suggested to supplement the research foundation of the author's team (such as previous research results in related fields), to enhance the credibility and persuasiveness of the research.

**********

6. PLOS authors have the option to publish the peer review history of their article (what does this mean?). If published, this will include your full peer review and any attached files.

If you choose “no”, your identity will remain anonymous but your review may still be made public.

**Do you want your identity to be public for this peer review?**  For information about this choice, including consent withdrawal, please see our Privacy Policy.

Reviewer #1: No

Reviewer #2: No

**********

[NOTE: If reviewer comments were submitted as an attachment file, they will be attached to this email and accessible via the submission site. Please log into your account, locate the manuscript record, and check for the action link "View Attachments". If this link does not appear, there are no attachment files.]

To ensure your figures meet our technical requirements, please review our figure guidelines: https://journals.plos.org/plosone/s/figures

You may also use PLOS’s free figure tool, NAAS, to help you prepare publication quality figures: https://journals.plos.org/plosone/s/figures#loc-tools-for-figure-preparation.

NAAS will assess whether your figures meet our technical requirements by comparing each figure against our figure specifications.

Attachment Submitted filename: renamed_65225.doc

# Author comment: Author response to Decision Letter 1

**DOI:** 10.1371/journal.pone.0354702.r002

12 Apr 2026

Response to the Editor and Reviewers

Dear Dr. Chen and Reviewers,

We sincerely thank you for your careful reading of our manuscript and for your constructive and insightful comments. In response to the comments received, we carried out a substantial revision of the manuscript. The revisions are concentrated in the Abstract, Introduction, the whole of Section 2 (Literature Review, including Sections 2.1–2.4), and the whole of Section 3 (Methods, including Sections 3.1–3.4). We also revised important parts of the Results section, including part of Section 4.1, parts of Sections 4.2 and 4.3, the whole of Section 4.4, and the final paragraph of Section 4.5. In the Discussion, we revised several sentences in Section 5.1 and made more substantial changes to Sections 5.2 and 5.3. In addition, the whole of Section 6 was revised, including Sections 6.1, 6.2, and 6.3, and we also updated the title page and substantially redrew Figure 1 together with its caption and source description, and redrew Figure 2 to clarify the theoretical and analytical framework. These changes were made to strengthen the alignment between the theoretical review and the research theme, improve methodological transparency and reproducibility, deepen the interpretation of the findings, ensure that the discussion remains proportionate to the evidence, and better comply with the journal’s editorial requirements. Below, we provide our point-by-point responses.

I. Response to the Academic Editor

1. Please ensure that your manuscript meets PLOS ONE’s style requirements, including file naming.

Response: Thank you for this reminder. We carefully checked the manuscript formatting and file naming conventions in accordance with the PLOS ONE style requirements and revised the submission files accordingly.

2. Please ensure that all author-generated code is shared in line with PLOS ONE’s code-sharing requirements.

Response: Thank you for this important reminder. We have reviewed the journal’s code-sharing policy and aligned the revised submission with the requirement that author-generated analytical code be made available in a way that facilitates reproducibility and reuse.

3. Please include additional information about your dataset in the Methods section and specify whether the collection and analysis complied with the terms and conditions of the data source.

Response: Thank you for this helpful comment. In response to this comment, we revised the Methods section to provide a more detailed description of the dataset and the data-processing procedure. Specifically, we clarified the data platforms, collection period, sample size, valid-review criteria, and main cleaning procedures. We also added a statement explaining that the study used only publicly accessible review pages, did not bypass access restrictions, and handled the data in anonymized and aggregated form for academic research purposes only. In addition, we clarified that the study was covered by a formal written ethics exemption issued by the relevant institutional ethics committee. These revisions were made to improve the transparency of the dataset description and to clarify the procedural and ethical context of the data collection and analysis.

4. The grant information in “Funding Information” and “Financial Disclosure” do not match. Please provide the correct grant numbers.

Response: Thank you for pointing this out. We carefully checked the funding information and prepared the corrected funding statement with the accurate grant numbers for inclusion in the cover letter and for updating the online submission system.

5. Please state what role the funders took in the study and include the amended Role of Funder statement in the cover letter.

Response: Thank you for this reminder. We prepared an amended Role of Funder statement and included it in the cover letter, in line with the journal’s request. If the funders had no role in study design, data collection and analysis, decision to publish, or preparation of the manuscript, we have stated this explicitly.

6. Funding information should not appear in the Acknowledgments section or other areas of the manuscript; please remove funding-related text from the manuscript.

Response: Thank you. We revised the manuscript accordingly and removed funding-related wording from the Acknowledgments section and other parts of the manuscript, retaining funding information only in the submission-related funding statement materials.

7. Please ensure that the corresponding author has a validated ORCID iD in Editorial Manager.

Response: Thank you for the reminder. We have checked this requirement and ensured that the corresponding author’s ORCID iD has been validated in Editorial Manager before resubmission.

8. The ethics statement should only appear in the Methods section.

Response: Thank you for this clarification. In response to this comment, we revised the manuscript so that the ethics statement now appears only in the Methods section, as required by the journal. Specifically, we incorporated the ethics-related description into the methodological account of data collection and anonymization, and removed repeated ethics-related wording from other parts of the manuscript. We also clarified that the study was covered by a formal written ethics exemption issued by the relevant institutional ethics committee, and that the online review data were handled in anonymized and aggregated form for academic research purposes only. These changes were made to comply with the journal’s formatting requirement and to present the ethical basis of the study more clearly and appropriately.

9. Figure 1 contains copyrighted images. Please either provide written permission under CC BY 4.0 or remove/replace the figure, and revise the caption accordingly.

Response: Thank you for pointing this out. In the revised manuscript, we redrew Figure 1. Specifically, we removed the satellite imagery, photographs, and other visual elements with potential copyright concerns, and replaced them with a cleaner and more readable map design. We also revised the figure caption and source information accordingly. These changes were intended to improve both the clarity of the figure and its compliance with the journal’s copyright and figure-preparation requirements.

II. Response to Additional Editor Comments

1.Strengthen the specificity of the case studies:

Add specific samples of Nanjing Tulou (such as Tianluogeng Tulou Group, Heikeng Tulou Group, etc.), clarify the basis for case selection (such as popularity, tourism development degree, and the preservation status of the living heritage), and enhance the representativeness and rigor of the research cases.

Response: Thank you for this valuable suggestion. In response, we revised Section 3.1 (Case Study Site) to strengthen the justification for selecting Nanjing Tulou as a representative living heritage case. Specifically, we added more concrete discussion of Tianluokeng, Yunshuiyao, and Hekeng, and clarified their different roles in terms of architectural heritage visibility, tourism development, and the preservation of living cultural practices. These additions were intended to improve the representativeness and specificity of the case study.

2. Refine the data collection methods and improve data transparency and reproducibility:

The summary only mentions "collecting tourist review content from Ctrip and Dianping over the past 15 years using Python 3.12.3", further details are needed: specify the exact time range of data collection (such as 2009-2024), the sample selection criteria (such as the definition of valid reviews: excluding advertisements, duplicates, and those without substantive content), the sample size, the ethical norms for data scraping (such as whether platform authorization was obtained, whether user information was anonymized), and the core code logic of Python scraping (which can be uploaded as supplementary materials), meeting the core requirements of PLOS ONE for the reproducibility of research methods.

Response: Thank you for this important comment. We revised Sections 3.2–3.4 to improve the transparency of data collection and preprocessing. Specifically, we clarified the time span of the dataset, the platform sources, the valid-review criteria, the final sample size, and the main cleaning procedures. We also added more explicit description of anonymization, aggregated reporting, and bias/representativeness considerations in Section 3.4, and aligned the manuscript with the repository-based disclosure of the anonymized minimal dataset and core analytical logic. These revisions were made to improve methodological transparency and reproducibility.

3. Supplement the rationality demonstration of the research methods, strengthen the adaptability of the methods, and improve the visual presentation of the theoretical framework: Clearly explain why the Service-Dominant Logic (SDL) framework was chosen as the research perspective, and elaborate on the adaptability of this framework to the "tourist perception, living heritage experience" and "culture-service symbiosis" model - such as the core viewpoint of SDL framework that "service is the core of value co-creation", how it supports the research core of "service as the medium for the transmission of living heritage culture", and how this framework provides theoretical support for the division of the tourist perception dimension and the analysis of the experience mechanism. At the same time, explain the advantages of combining ROSTCM6.0 software with Python (such as Python's efficient data scraping and preprocessing, ROSTCM6.0's precise text mining and sentiment analysis, and the collaborative improvement of research efficiency and analysis accuracy), as well as the division and correlation of the four methods: frequency word analysis, network semantic analysis, sentiment analysis, and dynamic topic modeling (DTM), avoiding method overloading, and clarifying the supporting role of each method for the research objectives (such as frequency word analysis identifying core perception points, network semantic analysis mining the correlation of perception dimensions, sentiment analysis quantifying tourist attitudes, and DTM tracking the dynamic changes in perception). Enhance the rigor of the research methods. Additionally, in the theoretical framework section, it is recommended to present the research logic of the paper in a clear graphical form, drawing a research framework diagram to visually display the complete logical chain of "theoretical basis (SDL framework) - research methods (multi-text analysis methods) - research content (tourist perception dimensions, sentiment tendencies, theme changes) - research conclusion (culture-service symbiosis model)", marking the relationships between each link, making the research logic clearer and more understandable.

Response: Thank you for this helpful suggestion. In response, we revised Sections 2.4 and 3.3, and redrew Figure 2, to clarify the rationale of the theoretical and methodological design. Specifically, we strengthened the explanation of why Service-Dominant Logic (SDL) is relevant to the present study, especially in relation to value co-creation, interaction-based experience formation, and the role of service in mediating cultural understanding in living heritage tourism. At the same time, we retained Experience Economy, Tourist Gaze, and Authenticity as complementary interpretive perspectives, so that the theoretical framework is more consistent with the overall logic of the manuscript. We also revised the methodological explanation to clarify the complementary roles of Python and ROST CM 6.0, and to distinguish more clearly the analytical functions of high-frequency word analysis, semantic network analysis, sentiment analysis, and Dynamic Topic Modeling (DTM). In addition, Figure 2 was redrawn to present the research logic more clearly by linking theoretical foundation, analytical support, multi-method text analysis, research content, and interpretive output. These revisions were made to improve the coherence, readability, and methodological transparency of the manuscript.

4. Strengthen the depth of result analysis, supplement the analysis of differences and mechanisms:

The existing results only present the dimensions of tourist perception, sentiment tendencies, and theme changes. Further details are needed for the analysis of differences: such as the differences in perception among tourists of different age groups and different tourist sources, the driving mechanisms of changes in tourist perception in different tourism development stages (such as policy guidance, media promotion, and infrastructure improvement). At the same time, for the core result of "service experience taking the leading position", supplement specific cases (such as specific problems in transportation and cost management or quality service cases), to enhance the persuasiveness of the results.

Response: Thank you for this insightful suggestion. In response, we revised Sections 4 and 5 to deepen the interpretation of the findings. Specifically, we expanded the discussion of the temporal evolution of tourist perception across different development stages, and linked these changes to broader contextual drivers such as heritage policy, media dissemination, infrastructure improvement, and service-related pressure in the mature stage. We also supplemented the discussion of the service-experience dimension with more concrete examples related to transportation, fee management, guided interpretation, and site management. Regarding subgroup differences, we carefully considered the suggestion; however, because reliable demographic information such as age group and tourist origin is not explicitly available in most online reviews, we did not conduct unsupported subgroup analysis. Instead, we addressed this issue more cautiously in Section 3.4 through an expanded discussion of data representativeness and bias control.

5. Improve the analysis of negative evaluations and enhance the practical value of the research:

The abstract only mentions three sources of negative evaluations (commercialization eroding cultural authenticity, insufficient transportation accessibility, and service management deficiencies), and further detailed analysis is needed: The specific manifestations of each negative evaluation (such as the specific forms of commercialization: over-commercialized stores, false cultural experiences, etc.; specific scenarios of insufficient transportation accessibility: external transportation outside the scenic area, internal connections, etc.), as well as the ranking of the influence degree of different negative factors, will provide support for proposing targeted improvement measures in the future.

Response: Thank you for this important suggestion. We revised the Results and Practical Implications-related discussion to deepen the analysis of negative evaluations. Specifically, we moved beyond the simple listing of three broad negative categories and further unpacked the concrete manifestations of over-commercialization, accessibility problems, and service-management-related dissatisfaction. We also used this more detailed negative-evaluation evidence to strengthen the practical implications proposed later in the manuscript, so that the recommendations are more directly grounded in the empirical findings.

6. Strengthen the innovation and rationality demonstration of the "culture-service symbiosis" model:

The "culture-service symbiosis" model proposed in the abstract is the core innovation point, and it is necessary to further supplement the specific connotation, constituent elements (such as specific indicators at the cultural level and service level), and operation mechanism of the model, clarify the differences and connections between this model and existing related theories (such as the SDL framework, cultural heritage protection theory), and demonstrate the innovation and feasibility of the model; at the same time, supplement the verification methods of the model (such as combining case data, interview data, etc.), to avoid the model being proposed too abruptly and enhance the theoretical contribution of the research.

Response: Thank you for this valuable comment. In response to this comme

# Peer review: Decision Letter 1

**DOI:** 10.1371/journal.pone.0354702.r003

**Authors:** Yile Chen

18 May 2026

PONE-D-26-04498R1

Tourist Perceptions and Living Heritage Experiences in Fujian Nanjing Tulou Traditional Villages Based on Social Media Data Analysis

PLOS One

Dear Dr. Wang,

Thank you for submitting your manuscript to PLOS ONE. After careful consideration, we feel that it has merit but does not fully meet PLOS ONE’s publication criteria as it currently stands. Therefore, we invite you to submit a revised version of the manuscript that addresses the points raised during the review process.

Please submit your revised manuscript by Jul 02 2026 11:59PM. If you will need more time than this to complete your revisions, please reply to this message or contact the journal office at plosone@plos.org. When you're ready to submit your revision, log on to https://www.editorialmanager.com/pone/ and select the 'Submissions Needing Revision' folder to locate your manuscript file.

Please include the following items when submitting your revised manuscript:

- A letter that responds to each point raised by the academic editor and reviewer(s). You should upload this letter as a separate file labeled 'Response to Reviewers'.
- A marked-up copy of your manuscript that highlights changes made to the original version. You should upload this as a separate file labeled 'Revised Manuscript with Track Changes'.
- An unmarked version of your revised paper without tracked changes. You should upload this as a separate file labeled 'Manuscript'.

If you would like to make changes to your financial disclosure, please include your updated statement in your cover letter. Guidelines for resubmitting your figure files are available below the reviewer comments at the end of this letter.

If applicable, we recommend that you deposit your laboratory protocols in protocols.io to enhance the reproducibility of your results. Protocols.io assigns your protocol its own identifier (DOI) so that it can be cited independently in the future. For instructions see: https://journals.plos.org/plosone/s/submission-guidelines#loc-laboratory-protocols. Additionally, PLOS ONE offers an option for publishing peer-reviewed Lab Protocol articles, which describe protocols hosted on protocols.io. Read more information on sharing protocols at https://plos.org/protocols?utm_medium=editorial-email&utm_source=authorletters&utm_campaign=protocols.

As the corresponding author, your ORCID iD is verified in the submission system and will appear in the published article. PLOS supports the use of ORCID, and we encourage all coauthors to register for an ORCID iD and use it as well. Please encourage your coauthors to verify their ORCID iD within the submission system before final acceptance, as unverified ORCID iDs will not appear in the published article. *Only*  the individual author can complete the verification step; PLOS staff *cannot*  verify ORCID iDs on behalf of authors.

We look forward to receiving your revised manuscript.

Kind regards,

Yile Chen, Ph.D. in Architecture

Academic Editor

PLOS One

Journal Requirements:

If the reviewer comments include a recommendation to cite specific previously published works, please review and evaluate these publications to determine whether they are relevant and should be cited. There is no requirement to cite these works unless the editor has indicated otherwise.

[Note: HTML markup is below. Please do not edit.]

Reviewers' comments:

Reviewer's Responses to Questions

**Comments to the Author**

1. If the authors have adequately addressed your comments raised in a previous round of review and you feel that this manuscript is now acceptable for publication, you may indicate that here to bypass the “Comments to the Author” section, enter your conflict of interest statement in the “Confidential to Editor” section, and submit your "Accept" recommendation.

Reviewer #1: All comments have been addressed

Reviewer #3: All comments have been addressed

**********

2. Is the manuscript technically sound, and do the data support the conclusions?

The manuscript must describe a technically sound piece of scientific research with data that supports the conclusions. Experiments must have been conducted rigorously, with appropriate controls, replication, and sample sizes. The conclusions must be drawn appropriately based on the data presented.

Reviewer #1: Yes

Reviewer #3: No

**********

3. Has the statistical analysis been performed appropriately and rigorously?

Reviewer #1: Yes

Reviewer #3: Yes

**********

4. Have the authors made all data underlying the findings in their manuscript fully available?

The PLOS Data policy requires authors to make all data underlying the findings described in their manuscript fully available without restriction, with rare exception (please refer to the Data Availability Statement in the manuscript PDF file). The data should be provided as part of the manuscript or its supporting information, or deposited to a public repository. For example, in addition to summary statistics, the data points behind means, medians and variance measures should be available. If there are restrictions on publicly sharing data—e.g. participant privacy or use of data from a third party—those must be specified.

Reviewer #1: Yes

Reviewer #3: Yes

**********

5. Is the manuscript presented in an intelligible fashion and written in standard English?

PLOS ONE does not copyedit accepted manuscripts, so the language in submitted articles must be clear, correct, and unambiguous. Any typographical or grammatical errors should be corrected at revision, so please note any specific errors here.

Reviewer #1: Yes

Reviewer #3: No

**********

6. Review Comments to the Author

Please use the space provided to explain your answers to the questions above. You may also include additional comments for the author, including concerns about dual publication, research ethics, or publication ethics. (Please upload your review as an attachment if it exceeds 20,000 characters)

Reviewer #1: Although the article has been revised, its overall innovation is insufficient. The core relies solely on textual statistics to draw important conclusions about service experience, and the subsequent theoretical and practical suggestions are quite abrupt. There are still several shortcomings:

(1) Shallow research: The overall focus is on data description and phenomenon induction, which only indicates the importance of services. Why service is important can be further analyzed qualitatively by combining practical cases behind some comments.

(2) Theoretical hollowing out: The concept of "culture service symbiosis" is proposed based solely on statistical evidence that services are important. To propose this conceptual model, there must be rigorous evidence in the text to support the scientific validity of the "dual symbiosis of culture and service".

(3) Suggestion for hollowing out: The practical measures tend to be metaphysical, and can be specified based on the results collected from the text and combined with examples of earthen buildings, which should be consistent with the previous text. At the same time, it is also necessary to demonstrate its feasibility and feasibility.

(4) Data singularity: The collected review data is limited to domestic tourism platforms, and the sample is concentrated within China, lacking evaluation and comparison from international tourists. The data coverage and representativeness are insufficient, and the research conclusions are limited.

Reviewer #3: 1. I recommend that the authors expand the theoretical framework by incorporating the conceptual distinction among place identity, place image, and place reputation (see for example Matlovičová 2024, Michalková 2014). This addition is highly relevant because the manuscript repeatedly discusses destination image, cultural authenticity, media-induced symbolism, tourist perception, and commercialization, but it does not sufficiently distinguish between the identity of Nanjing Tulou as a living heritage place, the image constructed by tourists through online reviews, and the broader reputation of the destination as a World Heritage attraction. This distinction would help the authors avoid conceptual overlap in the current manuscript, where “tourist perception,” “destination image,” “cultural authenticity,” and “heritage attractiveness” are sometimes used almost interchangeably. This framework is especially important for the Nanjing Tulou case. The place identity of Nanjing Tulou includes its rammed-earth architecture, Hakka settlement traditions, clan organization, everyday village life, landscape setting, and World Heritage status. The place image analyzed in this manuscript is not identical to that identity; it is the image reconstructed through tourist reviews on Ctrip and Dianping. This image is selective, subjective, and mediated by tourists’ expectations, film and animation narratives, service encounters, transportation experience, and commercialization. The place reputation of Nanjing Tulou, in turn, is formed through the accumulated and generalized public evaluation of the destination, including its reputation as an iconic World Heritage landscape, a cinematic tourism site, and possibly also an over-commercialized tourist attraction. The manuscript would therefore benefit from adding a short theoretical subsection. In this subsection, the authors could explain that the study does not directly measure the full identity of Nanjing Tulou, nor the totality of its reputation. Rather, it examines the tourist-generated place image reflected in online review texts. This image partially reflects the destination’s identity but is also shaped by selective perception, media influence, service quality, and personal experience. This distinction would make the research object much clearer.

2. The proposed Culture–Service Symbiosis framework is the most original part of the manuscript, but it is introduced too late and remains conceptually vague. The authors should define it clearly in the theoretical framework section, not only in the Discussion and Conclusion. The previous theoretical addition would also strengthen the proposed Culture–Service Symbiosis framework. Service can be interpreted as a mediator between place identity and place image: good interpretation, transparent management, and culturally sensitive services help visitors decode the identity of the place into a richer and more positive image. Conversely, poor service and excessive commercialization distort this transformation and may gradually weaken the destination’s reputation. The attached article’s emphasis on the dynamic, subjective, and processual nature of place image is particularly useful here, because the manuscript’s longitudinal social media design is precisely suited to tracing how place image changes over time .

3. At present, the framework seems to contain two mechanisms: Cultural decoding: services such as interpretation, guiding, signage, and digital tools help visitors understand the cultural meaning of Tulou architecture and Hakka life. Authenticity buffering: transparent, efficient, and culturally sensitive management reduces the negative impact of commercialization and service friction on perceived authenticity. This is a good starting point, but the manuscript must explain how these mechanisms are theoretically derived from Service-Dominant Logic, authenticity theory, and living heritage theory. SDL is mentioned, but core SDL concepts such as value co-creation, resource integration, service ecosystems, actors, institutional arrangements, and value-in-use are not sufficiently used. The authors should move beyond the general statement that “service matters” and show precisely how service mediates the relationship between heritage resources and visitor experience.

10. The manuscript should be more precise in describing the World Heritage property. “Fujian Tulou” is the World Heritage property, while Nanjing Tulou Scenic Area is one component or part of that wider property. The paper should avoid implying that Nanjing Tulou alone is the entire World Heritage site. The claim that UNESCO described Tianluokeng as “the pinnacle of geometric aesthetics in rammed-earth architecture” needs verification and citation. If this is not an official UNESCO phrase, it should be rephrased. The discussion of “Hakka culture” should also be more nuanced. Fujian Tulou are often associated with Hakka communities, but not all Tulou heritage can be reduced to a simplified Hakka cultural label. The authors should avoid essentializing local culture and should pay more attention to resident life, clan organization, ritual practice, and the relationship between tourism and community agency.

References:

Matlovičová, K. 2024. The Triadic Nexus: Understanding the Interplay and Semantic Boundaries Between Place Identity, Place Image, and Place Reputation. Folia Geographica 66(1), 69-102.

MICHALKOVÁ, J. (2014). Place branding as a phenomenon of a globalizing world: Theoretical background. Folia Geographica, 56(2), pp. 56-69.

**********

7. PLOS authors have the option to publish the peer review history of their article (what does this mean?). If published, this will include your full peer review and any attached files.

If you choose “no”, your identity will remain anonymous but your review may still be made public.

**Do you want your identity to be public for this peer review?**  For information about this choice, including consent withdrawal, please see our Privacy Policy.

Reviewer #1: No

Reviewer #3: No

**********

[NOTE: If reviewer comments were submitted as an attachment file, they will be attached to this email and accessible via the submission site. Please log into your account, locate the manuscript record, and check for the action link "View Attachments". If this link does not appear, there are no attachment files.]

To ensure your figures meet our technical requirements, please review our figure guidelines: https://journals.plos.org/plosone/s/figures

You may also use PLOS’s free figure tool, NAAS, to help you prepare publication quality figures: https://journals.plos.org/plosone/s/figures#loc-tools-for-figure-preparation.

NAAS will assess whether your figures meet our technical requirements by comparing each figure against our figure specifications.

# Author comment: Author response to Decision Letter 2

**DOI:** 10.1371/journal.pone.0354702.r004

22 Jun 2026

Response to Reviewers

Manuscript ID: PONE-D-26-04498R1

Title: Tourist Perceptions and Living Heritage Experiences in Nanjing Tulou Traditional Villages, Fujian, China: A Social Media Data Analysis

Journal: PLOS ONE

Dear Academic Editor and Reviewers, thank you for the constructive comments on our revised manuscript. We have prepared a further revision that strengthens the theoretical framework, clarifies the research object, adds more qualitative interpretation of the review evidence, corrects World Heritage wording, and more explicitly acknowledges the representativeness limits of the dataset. All changes are highlighted in the marked manuscript.

Response to Reviewer #1

Comment 1: The overall innovation is insufficient and the manuscript relies too heavily on textual statistics.

Response: We revised the manuscript to make the original contribution clearer and less dependent on descriptive statistics alone. The revised theoretical framework now distinguishes place identity, place image, and place reputation, and defines Culture-Service Symbiosis before the empirical sections. In the Discussion, the statistical results are interpreted with concrete comment-based cases such as Hekeng's perceived homogenization, inadequate interpretation, repeated ticket checks, transport friction, and commercialization. These changes appear in Sections 2.4, 2.5, 5.1, 5.2, and 5.3, and clarify that the paper's contribution is not only the frequency distribution of review terms, but the explanation of how service mediates the transformation from living heritage identity into tourist-generated place image. Manuscript location: Sections 2.4-2.5, lines 205-280; Sections 5.1-5.3, lines 1101-1227.

Comment 2: The analysis remains shallow; why service is important should be further analyzed qualitatively with practical cases.

Response: We added qualitative interpretation throughout the Discussion. The revised manuscript explains the Hekeng case as a cultural decoding failure: without guides, storytelling, signage, and route design, visitors tend to reduce distinct tulou histories and construction techniques to repetitive visual symbols. We also explain authenticity buffering through specific negative-review themes, including repeated ticket checks, unclear fees, congestion, and homogenized souvenir streets. These cases, now discussed mainly in Sections 5.1-5.3 and Appendix A, connect the text-mining results with the mechanisms behind service importance. Manuscript location: Sections 5.1-5.3, lines 1101-1227; Appendix A, lines 1317-1417.

Comment 3: The Culture-Service Symbiosis concept is theoretically hollow and requires rigorous support.

Response: We have moved the Culture-Service Symbiosis framework into the theoretical framework section and defined it explicitly. The revised framework is derived from SDL, authenticity theory, living heritage theory, and the place identity/image/reputation distinction. It identifies two mechanisms: cultural decoding, through which interpretation and guidance help visitors understand Tulou architecture and resident life; and authenticity buffering, through which transparent and culturally sensitive management reduces the negative effects of commercialization and service friction on perceived authenticity. These changes are made in Section 2.5 and further supported in Sections 5.2 and 6.1. Manuscript location: Section 2.5, lines 231-280; Section 5.2, lines 1142-1190; Section 6.1, lines 1233-1255.

Comment 4: Practical recommendations are too abstract and should be tied to the results and feasibility.

Response: We revised the practical implications to be more operational. The revised recommendations include unified ticketing and verification, transparent fee lists, time-slot and visitor-flow management, commercial zoning that protects residential and ritual spaces, locally grounded products and resident-led interpretation, shuttle and parking coordination, multilingual signage, trained guides, and short digital interpretation modules. These revisions appear in Section 6.3, and each recommendation is tied to a recurring issue identified in the corpus. Manuscript location: Section 6.3, lines 1266-1292.

Comment 5: The data are limited to domestic tourism platforms and lack international comparison.

Response: We added a supplementary international-facing platform triangulation step. Publicly visible English-language reviews on platforms such as Tripadvisor and Trip.com were read as contextual evidence rather than incorporated into the main quantitative corpus. The revision explains that this international-facing platform content also mentions architectural uniqueness, World Heritage value, transport difficulty, interpretation, and commercialization. It also clarifies why these data were not merged into the main statistical corpus: the pages mix international tourists, domestic users, platform-generated travel notes, and translated content, and they do not provide comparable full-period metadata. We therefore keep the main corpus unchanged while explicitly limiting the claims to Chinese-platform tourist-generated place image and identifying systematic multilingual comparison as a future research direction. These changes appear in Sections 3.4 and 6.4. Manuscript location: Section 3.4, lines 395-433; Section 6.4, lines 1293-1316.

Response to Reviewer #3

Comment 1: Add the distinction among place identity, place image, and place reputation.

Response: We added a new subsection, 'Place Identity, Place Image, and Place Reputation,' citing Matlovičová (2024) and Michalková (2014). The revised manuscript clarifies that the place identity of the wider Fujian Tulou heritage landscape includes rammed-earth architecture, settlement morphology, clan organization, resident everyday life, ritual and customary practices, mountain landscape settings, and World Heritage status. The study measures tourist-generated place image reconstructed from online review texts, not the full identity or total reputation of the destination. This revision appears in Section 2.4. Manuscript location: Section 2.4, lines 205-230.

Comment 2: Define Culture-Service Symbiosis earlier and connect it to the place image framework.

Response: We now define Culture-Service Symbiosis in the theoretical framework rather than only in the Discussion and Conclusion. The revised framework treats service as a mediating interface between place identity and tourist-generated place image. Good interpretation, transparent management, and culturally sensitive services help visitors decode heritage identity into richer place image, while poor service and excessive commercialization may distort this transformation and create reputational risk. This revision appears in Section 2.5 and is applied in Sections 5.2 and 6.1. Manuscript location: Section 2.5, lines 231-280; Section 5.2, lines 1142-1190; Section 6.1, lines 1233-1255.

Comment 3: Derive the mechanisms from SDL, authenticity theory, and living heritage theory, rather than simply saying service matters.

Response: We revised the SDL discussion to use core concepts including value co-creation, resource integration, service ecosystems, actors, institutional arrangements, and value-in-use. In the revised manuscript, residents, guides, managers, platform users, and tourists are treated as actors who integrate resources through ticketing, route management, interpretation, commercial zoning, and online evaluation. This provides the theoretical basis for the two mechanisms of cultural decoding and authenticity buffering. These revisions appear in Sections 2.5 and 5.2. Manuscript location: Section 2.5, lines 231-280; Section 5.2, lines 1142-1190.

Comment 4: Correct World Heritage wording, verify the UNESCO phrase, and nuance Hakka culture.

Response: We corrected the manuscript to state that 'Fujian Tulou' is the UNESCO World Heritage property and that the Nanjing Tulou Scenic Area is a component of that wider property. We removed the unverified wording that UNESCO described Tianluokeng as 'the pinnacle of geometric aesthetics in rammed-earth architecture' and replaced it with a more cautious description of Tianluokeng's distinctive four-round-one-square composition. We also revised essentializing references to Hakka culture by adding resident everyday life, clan organization, ritual and customary practices, foodways, market scenes, and community agency. These changes appear in Sections 3.1, 5.1, and 6.3. Manuscript location: Section 3.1, lines 282-316; Section 5.1, lines 1101-1141; Section 6.3, lines 1266-1292.

Additional language and clarity revisions

Response: We revised terminology throughout the manuscript to distinguish tourist perception, tourist-generated place image, destination reputation, cultural authenticity, and living heritage identity. We also made the limitations section more precise and clarified that the international-facing review scan is supplementary rather than part of the main statistical corpus. Manuscript location: Sections 2.4-2.5, lines 205-280; Section 3.4, lines 395-433; Sections 5.1-5.3, lines 1101-1227; Section 6.4, lines 1293-1316.

Sincerely,

The Authors

Attachment Submitted filename: PONE-D-26-04498R1_Response_to_Reviewers_revised.docx

# Peer review: Decision Letter 2

**DOI:** 10.1371/journal.pone.0354702.r005

**Authors:** Yile Chen

12 Jul 2026

Tourist Perceptions and Living Heritage Experiences in Nanjing Tulou Traditional Villages, Fujian, China: A Social Media Data Analysis

PONE-D-26-04498R2

Dear Dr. Wang,

We’re pleased to inform you that your manuscript has been judged scientifically suitable for publication and will be formally accepted for publication once it meets all outstanding technical requirements.

Within one week, you’ll receive an e-mail detailing the required amendments. When these have been addressed, you’ll receive a formal acceptance letter and your manuscript will be scheduled for publication.

An invoice will be generated when your article is formally accepted. Please note, if your institution has a publishing partnership with PLOS and your article meets the relevant criteria, all or part of your publication costs will be covered. Please make sure your user information is up-to-date by logging into Editorial Manager at Editorial Manager® and clicking the ‘Update My Information' link at the top of the page. For questions related to billing, please contact billing support.

If your institution or institutions have a press office, please notify them about your upcoming paper to help maximize its impact. If they’ll be preparing press materials, please inform our press team as soon as possible -- no later than 48 hours after receiving the formal acceptance. Your manuscript will remain under strict press embargo until 2 pm Eastern Time on the date of publication. For more information, please contact onepress@plos.org.

Kind regards,

Yile Chen, Ph.D. in Architecture

Academic Editor

PLOS One

Additional Editor Comments (optional):

Reviewers' comments:

Reviewer's Responses to Questions

**Comments to the Author**

1. If the authors have adequately addressed your comments raised in a previous round of review and you feel that this manuscript is now acceptable for publication, you may indicate that here to bypass the “Comments to the Author” section, enter your conflict of interest statement in the “Confidential to Editor” section, and submit your "Accept" recommendation.

Reviewer #1: All comments have been addressed

Reviewer #3: All comments have been addressed

**********

2. Is the manuscript technically sound, and do the data support the conclusions?

The manuscript must describe a technically sound piece of scientific research with data that supports the conclusions. Experiments must have been conducted rigorously, with appropriate controls, replication, and sample sizes. The conclusions must be drawn appropriately based on the data presented.

Reviewer #1: Yes

Reviewer #3: Yes

**********

3. Has the statistical analysis been performed appropriately and rigorously?

Reviewer #1: Yes

Reviewer #3: Yes

**********

4. Have the authors made all data underlying the findings in their manuscript fully available?

The PLOS Data policy requires authors to make all data underlying the findings described in their manuscript fully available without restriction, with rare exception (please refer to the Data Availability Statement in the manuscript PDF file). The data should be provided as part of the manuscript or its supporting information, or deposited to a public repository. For example, in addition to summary statistics, the data points behind means, medians and variance measures should be available. If there are restrictions on publicly sharing data—e.g. participant privacy or use of data from a third party—those must be specified.

Reviewer #1: Yes

Reviewer #3: Yes

**********

5. Is the manuscript presented in an intelligible fashion and written in standard English?

PLOS ONE does not copyedit accepted manuscripts, so the language in submitted articles must be clear, correct, and unambiguous. Any typographical or grammatical errors should be corrected at revision, so please note any specific errors here.

Reviewer #1: Yes

Reviewer #3: Yes

**********

6. Review Comments to the Author

Please use the space provided to explain your answers to the questions above. You may also include additional comments for the author, including concerns about dual publication, research ethics, or publication ethics. (Please upload your review as an attachment if it exceeds 20,000 characters)

Reviewer #1: The manuscript has been revised in accordance with the previous review comments, and the deficiencies of this study have been objectively illustrated in the research prospects. The overall quality meets the acceptance criteria, so acceptance is recommended.

Remaining issue: The sources of figures are not fully annotated. Taking the aerial photos of scenic spots in Figure 1 as an example, it is necessary to clearly identify whether these photos are independently taken by the research team or quoted from external websites. Self-shot photographs are preferred. If images are cited from other websites, formal citations must be supplemented.

Reviewer #3: The authors have responded adequately to all comments and have substantially improved the manuscript. I have no further comments or objections. I therefore recommend that the manuscript be accepted for publication.

**********

7. PLOS authors have the option to publish the peer review history of their article (what does this mean?). If published, this will include your full peer review and any attached files.

If you choose “no”, your identity will remain anonymous but your review may still be made public.

**Do you want your identity to be public for this peer review?**  For information about this choice, including consent withdrawal, please see our Privacy Policy.

Reviewer #1: **Yes:**  Yuhong Ding

Reviewer #3: No

**********

# Editor report: Acceptance letter

**DOI:** 10.1371/journal.pone.0354702.r006

**Authors:** Yile Chen

PONE-D-26-04498R2

PLOS One

Dear Dr. Wang,

I'm pleased to inform you that your manuscript has been deemed suitable for publication in PLOS One. Congratulations! Your manuscript is now being handed over to our production team.

At this stage, our production department will prepare your paper for publication. This includes ensuring the following:

* All references, tables, and figures are properly cited

* All relevant supporting information is included in the manuscript submission,

* There are no issues that prevent the paper from being properly typeset

You will receive further instructions from the production team, including instructions on how to review your proof when it is ready. Please keep in mind that we are working through a large volume of accepted articles, so please give us a few days to review your paper and let you know the next and final steps.

Lastly, if your institution or institutions have a press office, please let them know about your upcoming paper now to help maximize its impact. If they'll be preparing press materials, please inform our press team within the next 48 hours. Your manuscript will remain under strict press embargo until 2 pm Eastern Time on the date of publication. For more information, please contact onepress@plos.org.

You will receive an invoice from PLOS for your publication fee after your manuscript has reached the completed accept phase. If you receive an email requesting payment before acceptance or for any other service, this may be a phishing scheme. Learn how to identify phishing emails and protect your accounts at https://explore.plos.org/phishing.

If we can help with anything else, please email us at customercare@plos.org.

Thank you for submitting your work to PLOS ONE and supporting open access.

Kind regards,

PLOS ONE Editorial Office Staff

on behalf of

Dr. Yile Chen

Academic Editor

PLOS One
